# Mitochondrial genome of *Anaphalis lactea* from the Qinghai-Xizang Plateau: structural and evolutionary insights into alpine adaptation

**DOI:** 10.3389/fpls.2026.1885043

**Published:** 2026-08-06

**Authors:** Rui Wang, Wanting Wang, Fuhao Zhang, Yanfeng He, Yingfang Shen, Xuze Zhang

**Affiliations:** 1 College of Ecological Environment and Resources, Qinghai Minzu University, Xining, Qinghai, China; 2 Qinghai Provincial Key Laboratory of High-value Utilization of Characteristic Economic Plants, Xining, Qinghai, China; 3 Qinghai Provincial Biotechnology and Analytical Test Key Laboratory, Xining, China; 4 School of Pharmacy, Qinghai Minzu University, Xining, China

**Keywords:** adaptive evolution, Anaphalis lactea, mitochondrial genome, Qinghai-Xizang Plateau, RNA Editing

## Abstract

**Introduction:**

Plant mitochondrial genomes exhibit remarkable structural complexity and play important roles in environmental adaptation. To investigate the evolutionary dynamics of alpine plants, this study assembled and characterized the complete mitochondrial genome of *Anaphalis lactea*, an endemic medicinal species native to the Qinghai-Xizang Plateau.

**Methods:**

Using PacBio HiFi long-read sequencing data, the mitochondrial genome of *A. lactea* was *de novo* assembled and annotated. We analyzed the genomic structure, repetitive sequences, chloroplast-derived DNA transfer, RNA editing sites, and codon usage patterns. Furthermore, the evolutionary patterns were evaluated by integrating Ka/Ks ratios, PAML site models, and phylogenetic analyses.

**Results:**

The complete mitochondrial genome was 297,265 bp in length, with a GC content of 44.71%, and contained 34 protein-coding genes, 20 tRNA genes, and 3 rRNA genes. A total of 271 repetitive sequences were identified, indicating substantial structural plasticity in the mitochondrial genome. Chloroplast-derived fragments accounted for 1.68% of the genome and were mainly distributed in non-coding regions. A total of 572 RNA editing sites were predicted, all of which resulted in non-synonymous amino acid substitutions. Codon usage bias was relatively weak and appeared to be primarily driven by natural selection. Ka/Ks analysis indicated that most genes evolved under purifying selection, whereas *ccmFn* exhibited a relatively high Ka/Ks ratio.Furthermore, PAML analysis identified positively selected sites L285A, Y303R and T378F in the *ccmFc* gene. Within the Asteraceae phylogenetic tree, *A. lactea* shows a strongly supported sister-group relationship with the tribe Anthemideae (PP = 1.00, BS = 100). Although mitochondrial DNA sequences are highly conserved, RNA editing, by altering the physicochemical properties of respiratory chain complex proteins, acts together with localized positively selected sites and may facilitate long-term adaptation to high-altitude environments through mitochondrial functional optimization.

**Conclusions:**

This study provides the first complete mitochondrial genome report for *A. lactea* within the tribe Gnaphalieae. These findings provide valuable genomic resources and new insights into the adaptive mechanisms of alpine plants and the evolutionary relationships within Asteraceae.

## 1 Introduction

In the context of global warming, the Qinghai-Xizang Plateau has experienced a pronounced elevation-dependent warming trend, with a warming rate approximately twice the global average (Wang et al., 2024c). As one of the world’s largest alpine biomes, this region is highly sensitive to climate change. It also harbors a remarkably rich temperate flora, characterized by exceptionally high levels of endemism (Miehe et al., 2011; Li et al., 2014). To date, 12,058 seed plant species have been recorded in the region, of which 38.2% are endemic (Sun et al., 2025). Within this environmentally heterogeneous region with steep ecological gradients, the family Asteraceae has become one of the dominant taxa because of its broad ecological adaptability and high environmental tolerance (Liu et al., 2006). Notably, *Anaphalis*, the largest genus within the Asian tribe *Gnaphalieae*, has undergone extensive diversification across the Qinghai-Xizang Plateau (Nie et al., 2012; Meng et al., 2010).

*Anaphalis lactea* is a perennial herb endemic to the Qinghai-Xizang Plateau and widely used in traditional Tibetan medicine (Yu et al., 2024; Ren et al., 2008). Its altitudinal distribution ranges from 2600 to 4700 m, spanning gradients from subalpine to alpine zones. Prolonged exposure to multiple environmental stresses, including low temperature, hypoxia, and strong ultraviolet radiation, has made *A. lactea* a valuable plant resource for studying alpine plant adaptation (Lin, 1979; Yao et al., 2022). This species maintains high leaf UV reflectance across different altitudes and adapts to intense plateau radiation by regulating epicuticular wax deposition and alkane chain-length composition. These traits are jointly influenced by environmental and genetic factors (Li et al., 2019; Xu et al., 2025). Furthermore, these environmental stresses may continuously affect cellular energy metabolism, implying an important yet underexplored role for mitochondrial function in adaptive responses (Luo et al., 2013). However, previous studies have mainly focused on phenotypic adaptations, whereas the underlying molecular mechanisms, particularly those related to mitochondrial regulation, remain poorly understood.

As the central organelles of cellular energy metabolism, mitochondria play essential roles in ATP synthesis and redox homeostasis (Mackenzie and McIntosh, 1999; Casanova et al., 2023). The proteins encoded by mitochondrial genomes are primarily involved in respiratory chain assembly and electron transport (Barreto et al., 2022; Vercellino and Sazanov, 2022). Consequently, mitochondrial function is critical for plant adaptation to high-altitude environments, and the mechanisms underlying mitochondrial genomic variation and functional regulation warrant further investigation (Wu et al., 2025; Monteiro-Batista et al., 2025; Selinski et al., 2024).Although plant mitochondrial genomes generally exhibit low nucleotide substitution rates, they are characterized by relatively large genome sizes and complex structures, including abundant repetitive sequences, frequent structural rearrangements, and intracellular gene transfer events (Sloan et al., 2012; Gualberto and Newton, 2017). This high degree of structural dynamism and lineage-specific evolutionary variation makes plant mitochondrial genomes valuable resources for taxonomic and evolutionary studies (Wideman et al., 2020; Butenko et al., 2024). However, owing to their structural complexity and the limitations of earlier sequencing and assembly technologies, research on plant mitochondrial genomes has lagged behind that on chloroplast genomes (Štorchová and Krüger, 2024). In species-rich families such as Asteraceae, high-quality complete mitochondrial genome resources remain limited, restricting a comprehensive understanding of organellar evolution and phylogenetic relationships (Wang et al., 2024a; Fu et al., 2026).

Therefore, this study employed PacBio HiFi long-read sequencing to assemble and characterize the mitochondrial genome of *A. lactea*. Specifically, this study aimed to (1) characterize the genomic structure, repetitive sequences, inter-organellar DNA transfer, RNA editing sites, and codon usage patterns of the mitochondrial genome, (2) evaluate selective pressures and potential adaptive evolutionary signals in mitochondrial protein-coding genes; and (3) investigate the phylogenetic position and evolutionary characteristics of *A. lactea* within the tribe *Gnaphalieae*. This study provides the first mitochondrial genomic resource for *Gnaphalieae* and offers new insights into the evolution and environmental adaptation of alpine plants.

## 2 Materials and methods

### 2.1 Plant material, DNA extraction, and sequencing

Fresh young leaves of *A. lactea* were collected from Menyuan County, Haibei Tibetan Autonomous Prefecture, Qinghai Province, China (101°13′22.16″ E, 37°37′23.38″ N). The samples were immediately frozen in liquid nitrogen and stored at −80 °C until further use. Total genomic DNA was extracted using the CTAB method (Schenk et al., 2023). DNA concentration, purity, and integrity were assessed using a NanoDrop 2000 spectrophotometer (Thermo Fisher Scientific, USA), a Qubit v3.0 Fluorometer (Invitrogen, USA), and agarose gel electrophoresis, respectively. High-quality DNA samples were subsequently used for library construction and sequencing.

Following DNA fragmentation, high-quality sequencing libraries were constructed through DNA damage repair, end repair, adapter ligation, enzymatic digestion, and purification. Subsequently, HiFi sequencing was performed on the PacBio Sequel II platform to generate high-accuracy long-read data.

The generated HiFi reads were primarily used for mitochondrial genome assembly. *De novo* assembly was performed using PMAT2 v2.0.2 (Bi et al., 2024), and the resulting assembly graphs were visualized and manually curated using Bandage v0.8.1 (Wick et al., 2015). Finally, the HiFi reads were mapped back to the assembled sequences using minimap2 v2.28 (Li, 2018), and the assembly was polished with NextPolish to obtain the complete mitochondrial genome sequence.

### 2.2 Mitochondrial genome annotation

The annotation of protein-coding genes (PCGs), transfer RNAs (tRNAs), and ribosomal RNAs (rRNAs) was performed using PMGA (Liu et al., 2024), followed by manual curation to obtain the final annotation results. Open reading frames (ORFs) were identified utilizing the Open Reading Frame Finder (http://www.ncbi.nlm.nih.gov/gorf/gorf.html), with the minimum length threshold set to 300 bp. After filtering out redundant sequences and those overlapping with known genes, the remaining ORFs were aligned against the NCBI non-redundant (nr) protein database restricted to Viridiplantae (green plants). Functional annotation was assigned based on best BLAST hits. To ensure the utmost accuracy, all preliminary annotation results were systematically reviewed and manually curated. The mitochondrial genome map of *A. lactea* was generated and visualized employing the OGDRAW platform (Greiner et al., 2019).

### 2.3 Analysis of repetitive sequences

Three types of repetitive sequences, including simple sequence repeats (SSRs), tandem repeats (TSRs), and dispersed repeats (DSRs), were identified in the mitochondrial genome. SSRs were identified using MISA v1.0 (Beier et al., 2017) with the following parameters: 1–10, 2–5, 3–4, 4–3, 5–3, and 6–3. TSRs were detected using TRF v4.09 (Benson, 1999) with the parameters 2 7 7 80 10 50 2000 -f -d -m. DSRs were identified using BLASTN v2.10.1 (Altschul et al., 1990) with a word size of 7 and an E-value threshold of 1e−5. The results were further filtered to remove redundant sequences and exclude tandem repeats. The distribution of these repetitive sequences within the mitochondrial genome was visualized using Circos v0.69-5 (Zhang et al., 2013). The identified SSR sites were mapped to the genome annotation to analyze their distribution across coding regions, introns, and intergenic regions.

### 2.4 Codon usage identification and Prediction of RNA editing sites

Relative synonymous codon usage (RSCU) values were calculated to assess codon usage bias. All calculations were performed based on non-redundant coding sequences (uniq CDS), which were extracted from the raw data using a custom Perl script. The CUSP program (Sharp and Li, 1986) and CodonW v1.4.4 were employed to compute the GC1, GC2, GC3, GCall, and GC3s values, as well as the effective number of codons (ENC) for each gene. the GC12 value was calculated (Grote et al., 2005). Visualization of the results was conducted utilizing the R package ggplot2 (Wickham, 2011).

Potential RNA editing sites were predicted using the PmtREP online server (http://112.86.217.82:9919/#/tool/alltool/detail/336). The impact of codon changes induced by RNA editing on the hydrophilicity and hydrophobicity of the encoded amino acids was statistically analyzed.

### 2.5 Ka/Ks, *Pi* analysis and positively selected site analyses

To evaluate evolutionary selective pressures, homologous gene pairs were identified among the analyzed species. The homologous sequences were aligned using MAFFT v7.505 (Katoh and Standley, 2013). Subsequently, the non-synonymous substitution rate Ka, synonymous substitution rate Ks, and Ka/Ks ratio were calculated using KaKs Calculator v2.0 (Wang et al., 2010) based on the Modified Li-Wu-Luo method. In addition, homologous gene sequences from different species were globally aligned using MAFFT v7.505, and nucleotide diversity Pi for each gene was calculated using DnaSP v5.

Positive selection analysis was conducted using site models implemented in the CodeML program of the PAML package through EasyCodeML (Yang, 2007). Six models were applied: M0, M1a, M2a, M7, M8, and M8a. Among these, M1a, M7, and M8a were treated as null models, whereas M2a and M8 were used as alternative models to detect positive selection at specific codon sites. Likelihood ratio tests (LRTs) were performed by comparing log-likelihood values between nested models to assess statistical significance at *P* < 0.05. In addition, the Bayes Empirical Bayes method was applied to identify amino acid sites potentially subjected to positive selection (Yang et al., 2005). The amino acid sequence of the *ccmFc* protein was used as a query to predict its three-dimensional structure using the AlphaFold 3 server (Abramson et al., 2024), and the resulting structure file was downloaded in CIF format. Structural visualization was subsequently performed using PyMOL v2.5.0 to analyze the spatial distribution and conformational characteristics of specific amino acid residues within the protein structure.

### 2.6 Synteny analysis

Genome-wide alignments between the assembled sequence and comparative sequences were performed using nucmer in MUMmer (4.0.0beta2) with the maxmatch parameter to generate dot plots. In the resulting dot plots, the horizontal axis (x-axis) represents the assembled sequence, whereas the vertical axis (y-axis) represents the comparative sequences. Forward alignments are indicated by red lines, whereas reverse-complement alignments are indicated by blue lines.

### 2.7 Chloroplast-to-mitochondrion-DNA transfer

Homologous sequences between the chloroplast and mitochondrial genomes were identified using BLASTN v2.10.1 with an E-value threshold of 1e−5 and a minimum sequence identity of 70%. The identified inter-organellar DNA fragments were summarized and visualized using Circos v0.69–5 to illustrate their distribution. To further characterize the transfer of protein-coding genes, amino acid sequences from the chloroplast genome were mapped to the mitochondrial genome using TBLASTN (E-value < 1e−5), enabling the identification of chloroplast-derived coding sequences integrated into the mitochondrial genome.

### 2.8 Phylogenetic analysis

Phylogenetic analysis was conducted using the complete mitochondrial genome of *A. lactea* generated in this study along with those of 22 additional species retrieved from GenBank. A total of 32 single-copy orthologous genes were identified and concatenated into a sequence dataset using PhyloSuite v1.2.3 (Zhang et al., 2020). Multiple sequence alignments were generated using MAFFT v7.505, and poorly aligned or highly divergent regions were removed using trimAl v1.4 (Capella-Gutiérrez et al., 2009) and Gblocks. The optimal nucleotide substitution model for the trimmed sequence matrix was determined using ModelFinder.

Maximum likelihood (ML) analysis was performed using IQ-TREE v2.2.0 (Nguyen et al., 2015), with node support assessed using 1,000 ultrafast bootstrap replicates. Bayesian inference (BI) analysis was also conducted using MrBayes v3.2.7 (Ronquist and Huelsenbeck, 2003). Two independent Markov chain Monte Carlo (MCMC) runs, each consisting of four chains, were performed for 3,000,000 generations, with the initial 25% of sampled trees discarded as burn-in. Convergence was considered achieved when the effective sample size (ESS) exceeded 200 and topological stability was attained.

## 3 Results

### 3.1 Annotation and structural analysis of the mitochondrial genome of *A. lactea*

**Figure 1:**
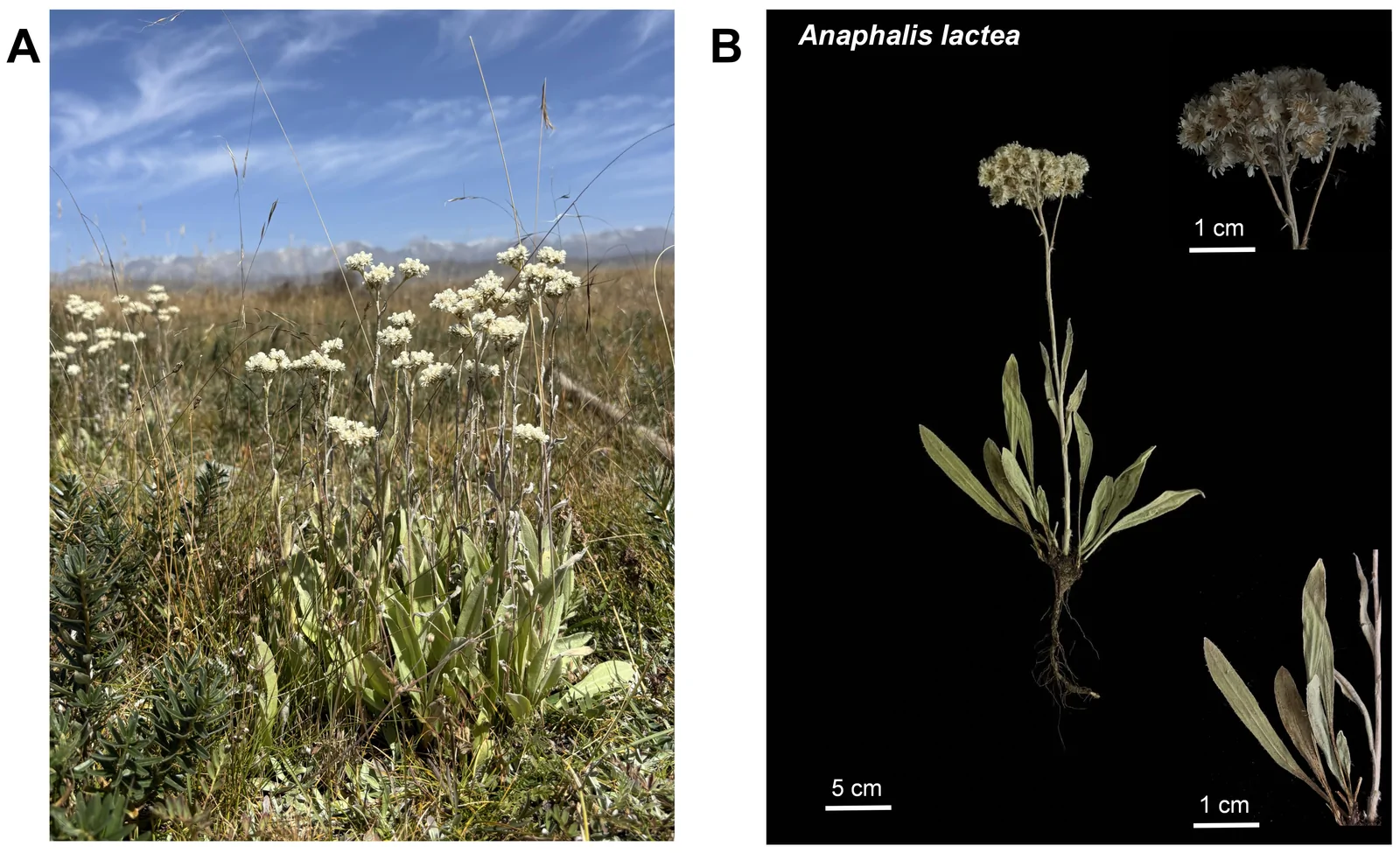
Habitat **(A)** and herbarium specimen **(B)** of *A. lactea*.

**Figure 2:**
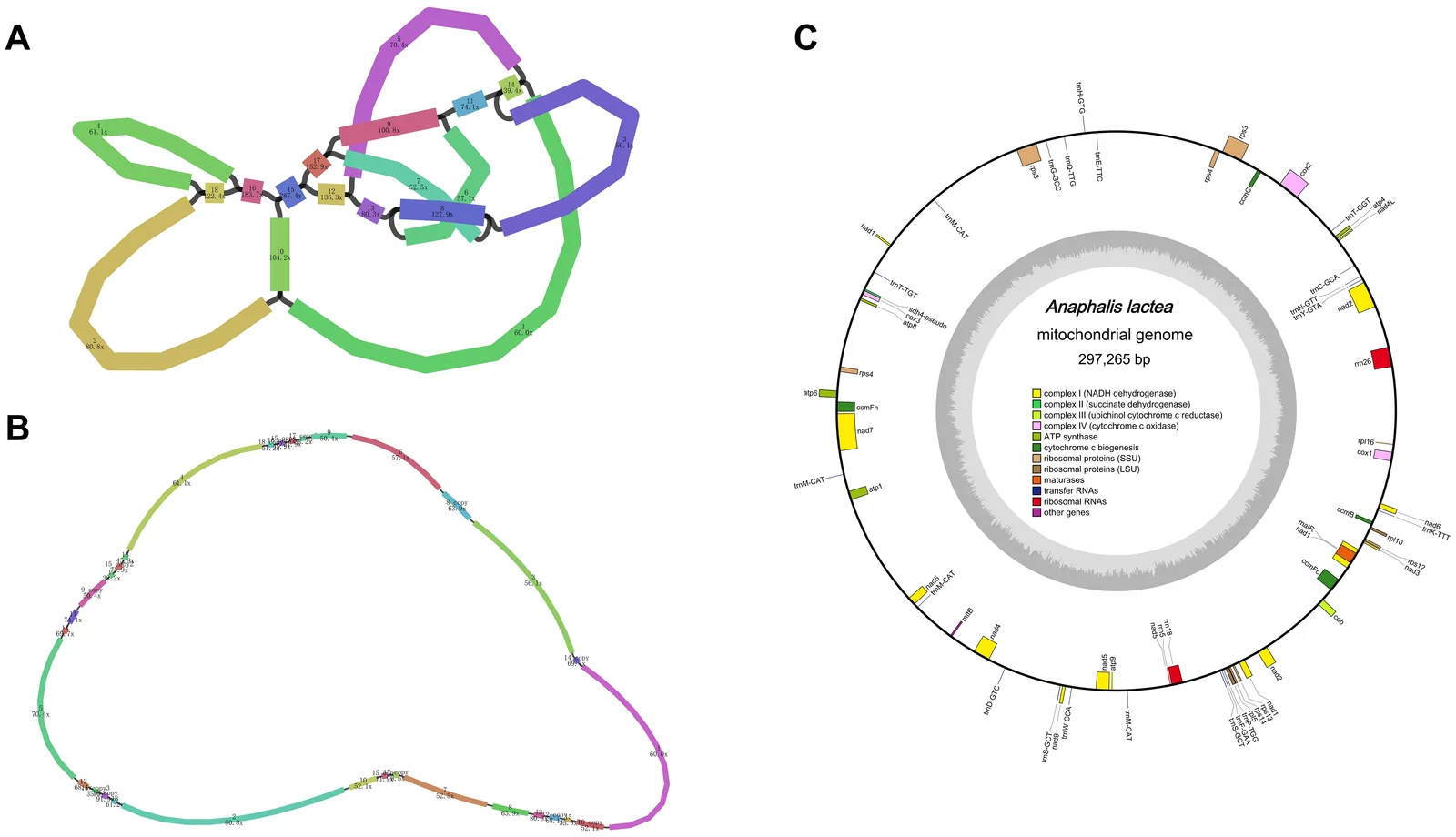
Assembly graphs and genome map of the *A. lactea* mitogenome. **(A)** Raw assembly graph. **(B)** Disentangled assembly graph. **(C)** Genome map of the mitogenome. Different gene types are distinguished using different colored blocks.

*A. lactea* utilized in the present study is a perennial herb widely distributed across the Qinghai-Xizang Plateau (**Figures 1A, B**), which also shows its natural habitat and specimen morphology. Whole-genome sequencing of the mitochondrial genome generated a total of 9.46 Gb of raw data, comprising 490,908 reads with a mean read length of 19,276 bp and an N50 read length of 19,703 bp. Following assembly with PMAT2, the resulting contigs were visually inspected using Bandage. After repetitive regions were filtered, a single putative circular chromosome was identified in the mitochondrial genome of *A. lactea* (**Figures 2A, B**). The complete mitochondrial genome is 297,265 bp in length with a GC content of 44.71%. Genome annotation identified a total of 58 genes. These genes include 34 protein-coding genes with a total length of 31,926 bp, accounting for 10.74% of the genome. The genome also contains three rRNA genes spanning 5,513 bp and accounting for 1.85% of the genome, as well as 20 tRNA genes spanning 1,489 bp and accounting for 0.50% of the genome (**Figure 2C**; **Table 1**).

**Table 1:** Summary of the *A. lactea* mitochondrial genome.

Parameter	Value
Total Length (bp)	297,265
GC%	44.71%
Number of PCGs	34
Number of rRNA genes	3
Number of tRNA genes	20
Total length of PCGs (bp)	31,926 (10.74%)
Total length of rRNAs (bp)	5,513 (1.85%)
Total length of tRNAs (bp)	1,489 (0.50%)

Functional annotation revealed that the mitochondrial genome contains genes associated with energy metabolism and protein synthesis. Specifically, these include three cytochrome c oxidase subunit genes, *cox1*, *cox2*, and *cox3*; four cytochrome c biogenesis genes, *ccmB*, *ccmC*, *ccmFc*, and *ccmFn*; five ATP synthase subunit genes, *atp1*, *atp4*, *atp6*, *atp8*, and *atp9*; nine NADH dehydrogenase subunit genes, *nad1*–*nad7*, *nad9*, and *nad4L*; and one cytochrome b gene, *cob*. In addition, eight ribosomal protein genes involved in translation were identified, including five small-subunit genes, *rps3*, *rps4*, *rps12*, *rps13*, and *rps14*, as well as three large-subunit genes, *rpl5*, *rpl10*, and *rpl16*. The genome also contains one maturase gene, *matR*, one membrane transport-related gene, *mttB*, and one pseudogene (**Supplementary Table 1**).

Although most mitochondrial genes were present as single copies, several duplicated genes were also identified. Among the protein-coding genes, two copies each of the ribosomal protein genes *rps3* and *rps4* were identified. Among the tRNA genes, four copies of *trnM-CAT* and two copies of *trnS-GCT* were identified, whereas the remaining tRNAs were present as single copies. The longest gene was *nad5*, with a length of 2,013 bp encoding 671 amino acids, whereas the shortest was *atp9*, with a length of 225 bp encoding 75 amino acids.

A total of eight protein-coding genes contained introns. Specifically, *nad1*, *nad2*, *nad5*, and *nad7* each contained four introns; *cox2* contained two introns; *nad4* and *ccmFc* each contained one intron; and both copies of *rps3* harbored one intron. Among the tRNA genes, only *trnT-TGT* contained an intron, whereas the remaining tRNAs were intronless.

### 3.2 Analysis of repetitive sequences

**Figure 3:**
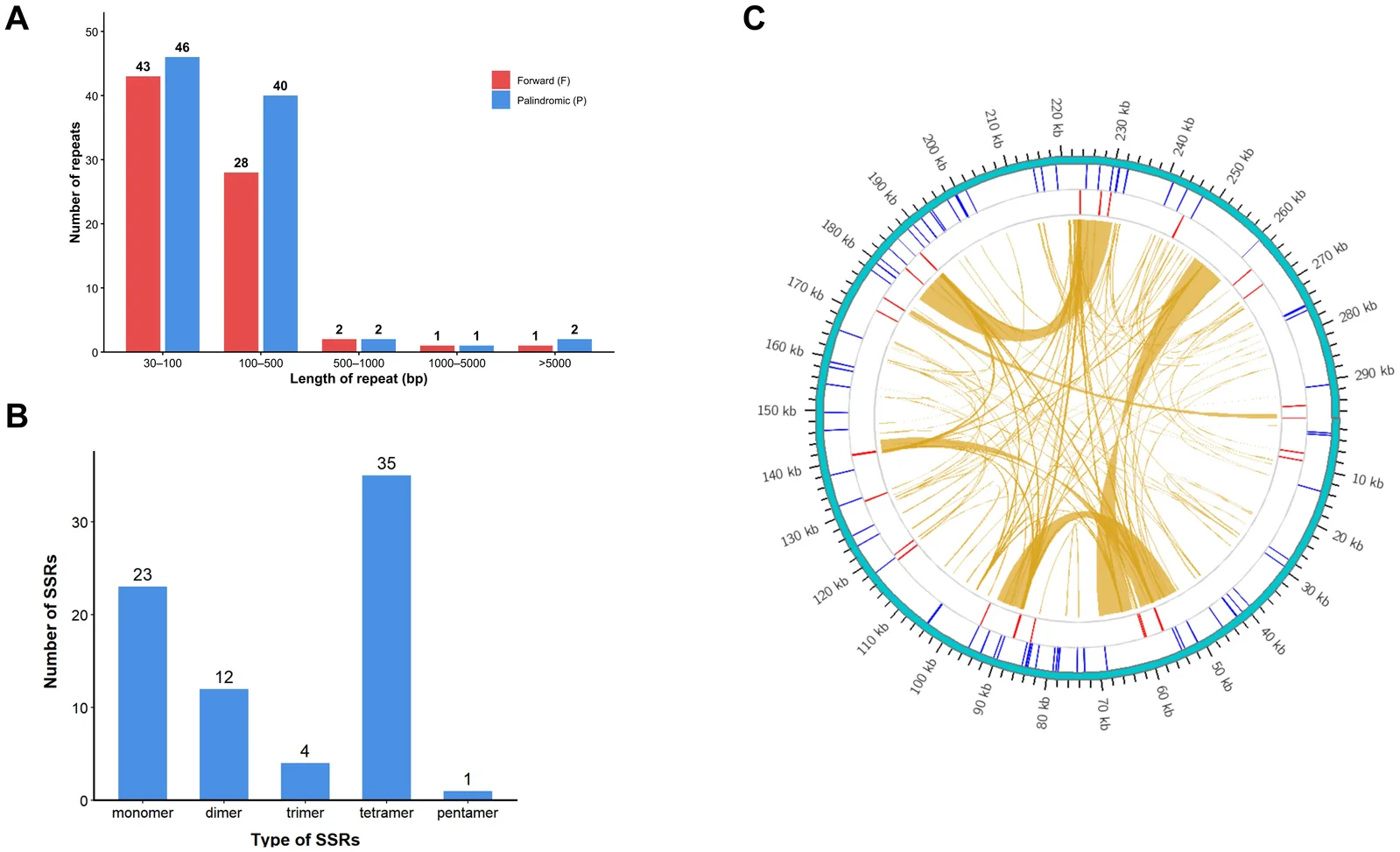
Repeat elements identified in the *A. lactea* mitogenome. **(A)** Frequency of identified simple sequence repeats (SSRs). **(B)** Size distribution of repeat sequences. **(C)** Distribution of repeat elements in the mitogenome. The outermost circle represents the mitogenome sequence, followed inward by simple sequence repeats (SSRs), tandem repeats, and dispersed repeats, respectively.

A total of 271 repetitive sequences were identified in the mitochondrial genome of *A. lactea*, including 75 simple sequence repeats, 30 tandem repeats, and 166 dispersed repeats. Among the dispersed repeats, 91 palindromic repeats and 75 forward repeats were identified. The lengths of these DSRs ranged from 30 to 8,367 bp, with an average length of 271 bp. Notably, 94.6% of the repeats were shorter than 500 bp, and repeats ranging from 30 to 100 bp represented the largest proportion at 53.6%. Furthermore, five long repeats exceeding 1 kb were detected, three of which were longer than 5 kb, with the longest reaching 8,367 bp (**Figure 3A**).

Based on repeat unit length, the 75 identified SSRs were classified into five categories. Tetranucleotide repeats were the most abundant (35 loci, 46.67%), followed by mononucleotide (23 loci, 30.67%), dinucleotide (12 loci, 16.00%), trinucleotide (4 loci, 5.33%), and pentanucleotide repeats (1 locus, 1.33%) (**Figure 3B**). Nucleotide composition analysis revealed that the repeat motifs exhibited a strong A/T bias. Among the mononucleotide repeats, A/T motifs accounted for 86.96% with 20 sites, whereas C/G motifs represented only 13.04% with three sites. In addition, 27 repeats composed exclusively of A/T bases were identified, accounting for 36.00% of all SSRs, including 20 mononucleotide, five dinucleotide, and two tetranucleotide repeats. Distribution analysis indicated that most simple sequence repeats were located in genic regions, with 68 sites accounting for 90.67%, whereas only seven sites representing 9.33% were distributed in intergenic spacers (IGS) (**Supplementary Table 2**).

In addition, 30 tandem repeats were detected, with repeat unit lengths ranging from 12 to 71 bp and sequence similarities ranging from 72% to 100%. Among these tandem repeats, 27 were located in genic regions and three in intergenic regions, with most repeats, totaling 24 sites, clustered within the nad2 gene region. Overall, the mitochondrial genome of *A. lactea* contains abundant repetitive sequences, predominantly characterized by short repeats and A/T-rich simple sequence repeats, which may contribute to genome structural variation and evolutionary dynamics (**Figure 3C**).

### 3.3 Analysis of RNA editing sites

**Figure 4:**
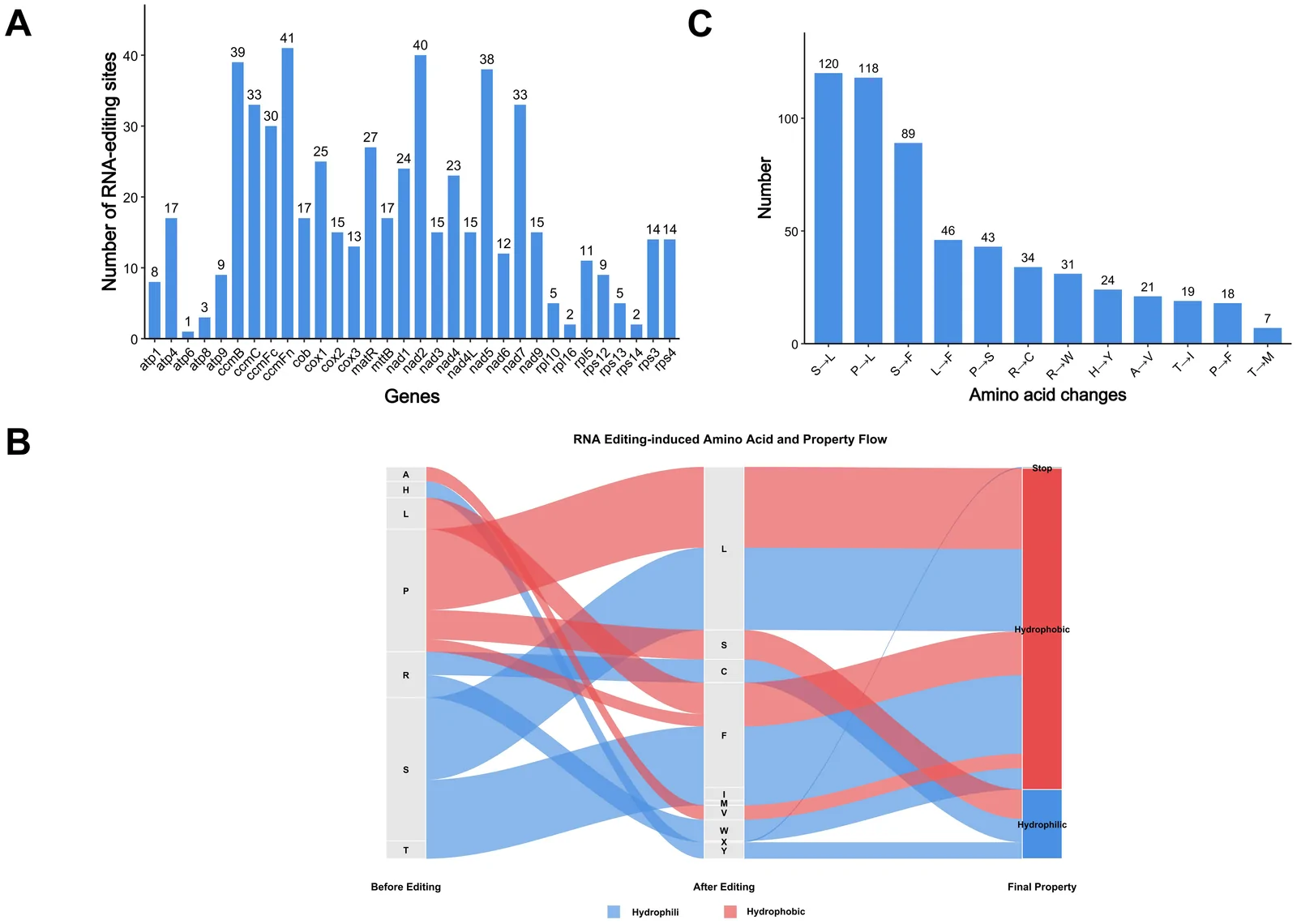
RNA editing analysis of protein-coding genes in the *A. lactea* mitogenome. **(A)** Distribution of RNA editing sites across protein-coding genes (PCGs). **(B)** Amino acid conversions induced by RNA editing in PCGs. **(C)** Sankey diagram showing the changes in amino acid properties before and after RNA editing.

A total of 572 RNA editing sites were identified in 32 protein-coding genes of the mitochondrial genome of *A. lactea*. Among these genes, the NADH dehydrogenase genes contained the largest number of editing sites, with 215 sites accounting for 37.6% of the total. Cytochrome c biogenesis genes contained 143 editing sites accounting for 25.0%, followed by ribosomal protein genes with 62 sites representing 10.8%, cytochrome c oxidase genes with 53 sites representing 9.3%, ATP synthase genes with 38 sites representing 6.6%, and other functional genes, including *cob*, *matR*, and *mttB*, with 61 sites representing 10.7% (**Figure 4A**). Notably, all predicted RNA editing events resulted in nonsynonymous substitutions, whereas no synonymous editing events were detected. These results suggest that RNA editing primarily influences protein function through alterations in amino acid sequences.

Analysis of codon position distribution showed that most editing events occurred at the second codon position, with 383 sites accounting for 66.96%, followed by the first codon position with 189 sites accounting for 33.04%, whereas no editing events were detected at the third codon position. RNA editing generated diverse amino acid substitutions, among which Ser-to-Leu and Pro-to-Leu conversions were the most frequent, occurring 120 and 118 times, respectively, followed by Ser-to-Phe conversions with 89 occurrence (**Figure 4B**).

A total of 563 edited sites were further analyzed to evaluate changes in amino acid properties. Conversions from hydrophilic to hydrophobic amino acids accounted for the largest proportion of editing events, representing 47.25% with 266 sites. In addition, 252 edited sites representing 44.76% retained their original properties, whereas 43 sites representing 7.64% changed from hydrophobic to hydrophilic. Furthermore, only two editing events representing 0.36% generated termination codons (**Supplementary Table 3**; **Figure 4C**).

### 3.4 Codon usage bias analysis

**Figure 5:**
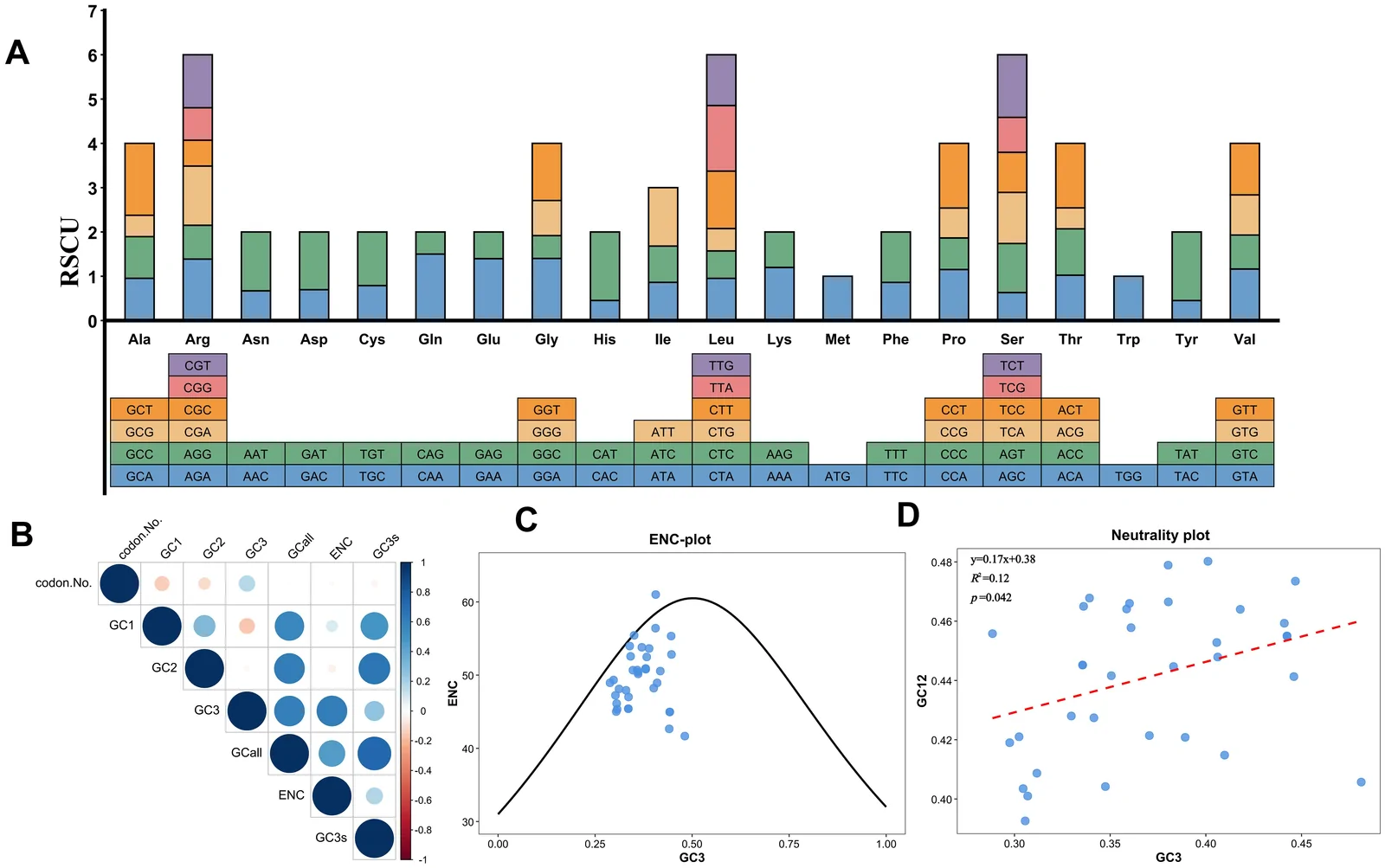
Codon usage bias of the *A. lactea* mitogenome. **(A)** RSCU bar plot. Lower blocks: codons per amino acid; bar height: sum of RSCU values. **(B)** Correlation of GC content and ENC. **(C)** ENC-plot. Black solid line: theoretical curve under mutational pressure. **(D)** Neutrality plot. Red dashed line: linear regression.

A total of 9,748 codons were identified in the mitochondrial genome of *A. lactea*, encoding all 20 standard amino acids and representing 64 codon types (**Supplementary Table 4**). Among these codons, UUU encoding phenylalanine was the most frequently used. In terms of amino acid composition, leucine was the most abundant amino acid, with 1,046 codons accounting for 10.73% of the total, followed by serine with 921 codons representing 9.45%. In contrast, cysteine was the least abundant amino acid, with only 137 codons accounting for 1.41% of the total. Relative synonymous codon usage analysis revealed that 30 codons exhibited RSCU values > 1, among which GCU encoding alanine showed the highest value, indicating a strong codon usage preference. The RSCU values of AUG encoding methionine and UGG encoding tryptophan were both 1, indicating no apparent codon usage bias. The remaining codons exhibited RSCU values < 1, indicating relatively low usage frequencies (**Figure 5A**).

Analysis of nucleotide composition in the *A. lactea* mitochondrial genome showed clear positional variation in GC content among codon sites (**Figure 5B**). The average GC content followed the order GC_1_ (47.61%) > GC_2_ (43.07%) > GC__all_ (42.50%) > GC_3_ (36.81%). To further investigate the mechanisms underlying codon usage bias, ENC plot and neutrality plot analyses were performed. ENC plot analysis showed that 26 genes representing 81.25% of the total were distributed below the expected curve, indicating that their observed ENC values were lower than those predicted under mutation pressure alone (**Figure 5C**). Neutrality plot analysis revealed no significant linear correlation between GC12 and GC3, with a regression coefficient of −0.064 and an R² value of 0.012, suggesting that variation in GC3 had little influence on GC12 (*P* > 0.05). These results further support the limited contribution of mutation pressure to codon usage bias (**Figure 5D**). In addition, the ENC values of individual genes ranged from 36.61 to 61.00, with an average value of 53.2. Furthermore, 24 genes representing 75% of the total exhibited ENC values greater than 50, indicating that the overall codon usage bias was weak and primarily shaped by natural selection rather than mutation pressure (**Supplementary Table 5**).

### 3.5 Selective pressure and molecular adaptation analysis

**Figure 6:**
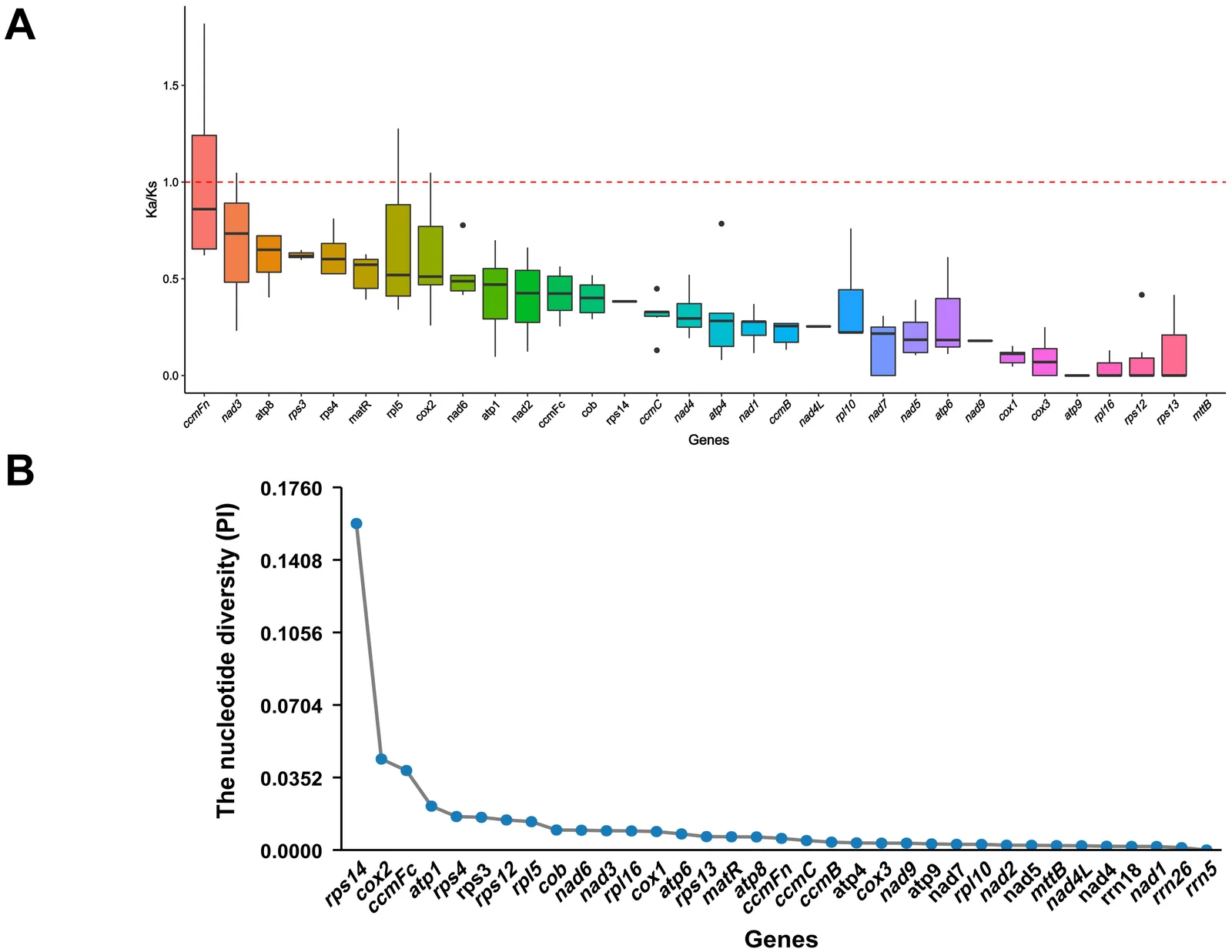
Evolutionary analysis of protein-coding genes in the *A. lactea* mitogenome compared with seven Asteraceae species. **(A)** Boxplot showing the distribution of Ka/Ks ratios for 32 PCGs between *A. lactea* and seven other Asteraceae species. **(B)** Line chart showing the nucleotide diversity **(Pi)** values of the PCGs.

Owing to the limited availability of mitochondrial genomic resources for the genus *Anaphalis* and the tribe *Gnaphalieae*, the mitochondrial genome of *A. lactea* was assembled and characterized in this study. Consequently, six representative species from the family Asteraceae, including *Chrysanthemum indicum*, *Arctium lappa*, *Dolomiaea costus*, *Lactuca sativa*, *Duhaldea cappa*, and *Helianthus annuus*, were selected to calculate evolutionary parameters for shared genes. Analysis of Ka/Ks ratios revealed that most mitochondrial PCGs exhibited Ka/Ks ratios below 1, indicating that they were primarily subjected to purifying selection during evolution (**Figure 6A**). Notably, genes such as *cox1*, *cox3*, *atp9*, *mttB*, and several ribosomal protein genes, including *rpl16*, *rps12*, and *rps13*, exhibited Ka/Ks values approaching 0, reflecting a high degree of sequence conservation. In contrast, the cytochrome c maturation-related gene *ccmFn* exhibited an average Ka/Ks ratio of 1.0191, with values exceeding 1 in several interspecific comparisons, suggesting an accelerated rate of sequence evolution. Furthermore, several genes, including *rpl5* (0.6726), *nad3* (0.6711), *rps4* (0.6253), and *atp8* (0.6064), ranked among the highest protein-coding genes in terms of average Ka/Ks ratios. Although these values were below 1, they indicate relatively faster evolutionary rates compared with those of other mitochondrial genes.

Sequence polymorphism analysis demonstrated substantial variation among different genes (**Figure 6B**). Specifically, the *rps14* gene exhibited the highest Pi value at approximately 0.15, which was markedly higher than those of the other genes. Furthermore, genes such as *cox2*, *ccmFc*, and *atp1* also exhibited relatively high Pi values, indicating their potential utility as molecular markers for species identification and population genetic analyses. Conversely, most NADH dehydrogenase subunit genes, including *nad4L* and *nad4*, as well as ribosomal RNA genes including *rrn18*, *rrn26*, and *rrn5*, exhibited Pi values approaching zero, reflecting strong sequence conservation.

**Figure 7:**
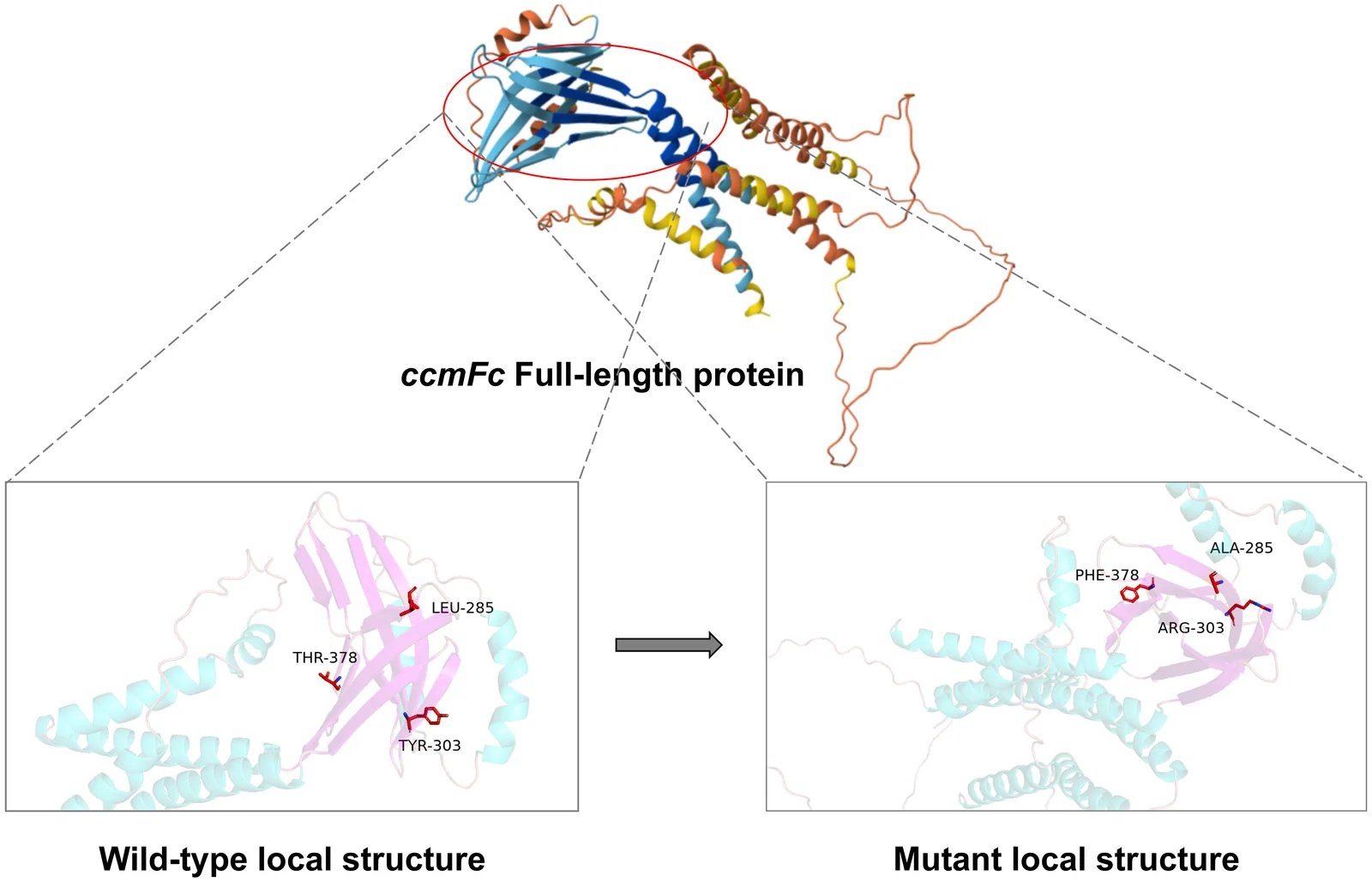
Comparison of the 3D protein structures between wild-type and mutant *ccmFc*. The upper panel shows the full-length protein model, and the lower panels highlight the local structural changes and specific amino acid substitutions. Purple represents β-sheets, blue represents α-helices, and orange lines represent random coils.

Given that both *ccmFn* and *ccmFc* are components of the cytochrome c maturation system and are involved in cytochrome *c* assembly and mitochondrial electron transport chain function, site models in PAML were used to analyze selection pressure acting on these two genes. Likelihood ratio tests for *ccmFc* were significant at *P* < 0.001 for comparisons between models M2a and M1a, M8 and M7, and M8 and M8a, indicating substantial selective heterogeneity among codon sites within this gene. Subsequent Bayes Empirical Bayes analysis under the M2a and M8 models identified three positively selected sites, namely 285L, 303Y, and 378T, within the *ccmFc* gene, suggesting that these loci may have undergone adaptive evolution (**Table 2**). In contrast, no significant positive selection signals were detected for the *ccmFn* gene in the PAML site model analysis (**Supplementary Table 6**). To further visualize the effects of selective pressure at the protein level, a three-dimensional structural model of the *ccmFc* protein was constructed (**Figure 7**). Structural comparison revealed that these positively selected sites, along with the corresponding amino acid substitutions (L285A, Y303R, and T378F), are located in the pink β-sheet within the core functional region. Compared with the wild type, the mutant exhibited significant localized conformational changes in this region.

**Table 2:** Selection pressure analysis of the mitochondrial *ccmFc* gene in *A. lactea* using site models in PAML CodeML.

Model	Parameters	lnL	Parameter estimates	Model comparison	LRT P-value	Positively selected sites (BEB)
M0	13	-1758.840046	*ω* = 0.59332			
M3	17	-1739.438764	*p0* = 0.04298,*ω0* = 0.16982	M3&M0	0.000000077	--
			*p1* = 0.81288,*ω1* = 0.16983			
			*p2* = 0.14414,*ω2* = 5.24295			
M1a	14	-1746.985484	*p0* = 0.58130,*ω0* = 0.41870			
			*p1* = 0.00000,*ω1* = 1.00000			
M2a	16	-1739.438764	*p0* = 0.85586,*ω0* = 0.16983	M2a&M1a	0.000527839	285L:0.981;303Y:0.965(*)
			*p1* = 0.00000,*ω1* = 1.00000			
			*p2* = 0.14414,*ω2* = 5.24296			
M7	14	-1747.990146	*p* = 0.00573,*q* = 0.01637			
M8	16	-1739.439092	*p0* = 0.85600,*p* = 20.41868,*q* = 99.00000			
			*p1* = 0.14400,*ω* = 5.24529			
M8a	15	-1746.985464	*p0* = 0.58130,*p* = 0.00500,*q* = 1.36109	M8&M7	0.000193341	285L:0.991;303Y:0.983;378T:0.959(*)
			*p1* = 0.41870,*ω* = 1.00000	M8&M8a	0.000102356	285L:0.991;303Y:0.983;378T:0.959(*)

### 3.6 Synteny and structural variation analysis of mitochondrial genomes

**Figure 8:**
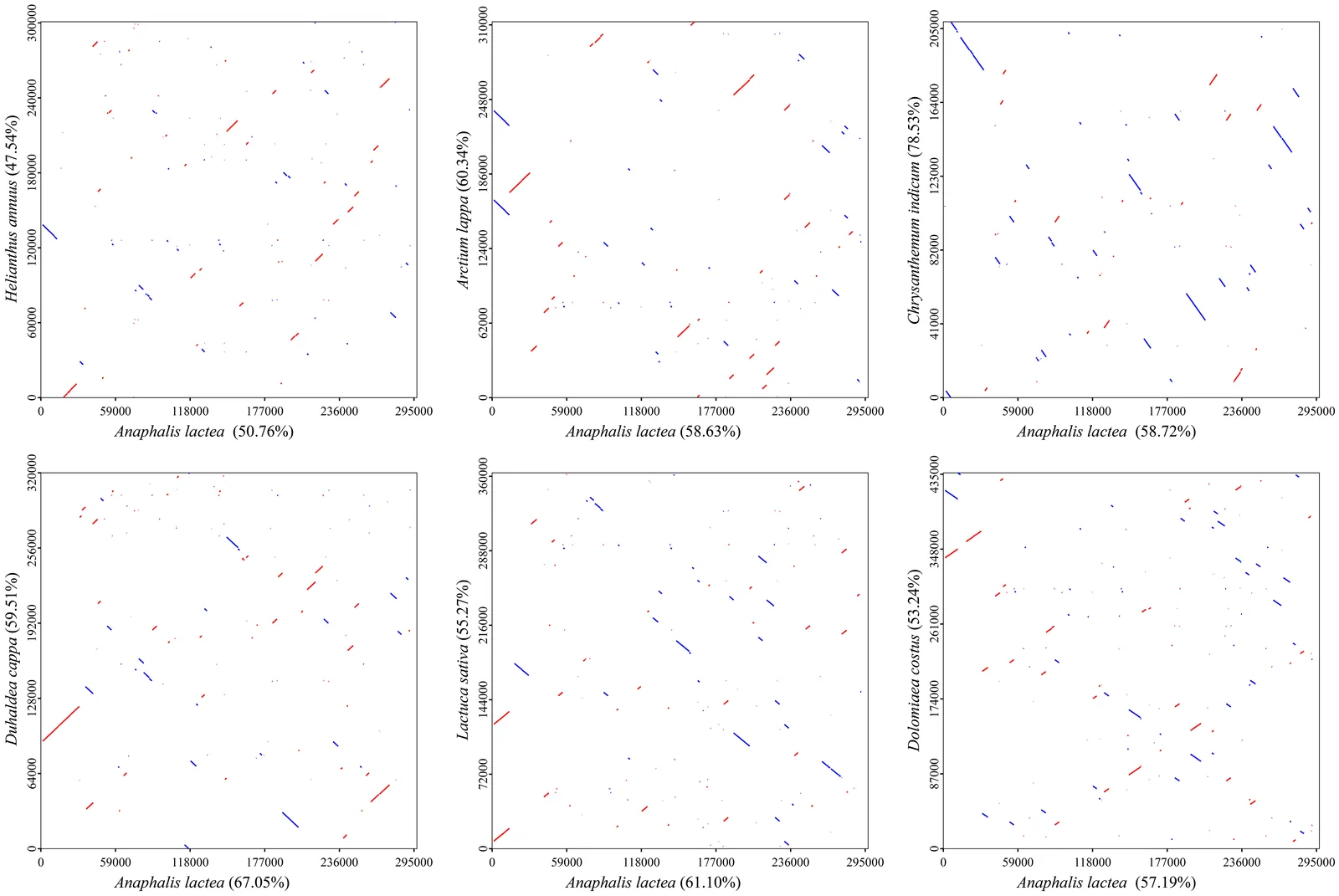
Synteny analysis of mitochondrial sequences. In each panel, the x-axis represents the assembled sequence (*A. lactea*), and the y-axis represents the query sequence from another species. The values in parentheses indicate the proportion of homologous sequences relative to the total genome size of the query species. Red lines indicate forward matches, and blue lines indicate reverse complementary matches.

Synteny analysis revealed multiple homologous blocks between the mitochondrial genome of *A. lactea* and those of six other Asteraceae species (**Figure 8**). Using *A. lactea* as the reference genome, the total length of the homologous regions ranged from 150,906 to 199,311 bp. These regions accounted for 50.76% to 67.05% of the entire genome length. These results indicate a moderate level of sequence conservation among the examined species. Among all compared species, the homologous region with *D. cappa* was the longest, reaching 199,311 bp and accounting for 67.05% of the genome. In contrast, the homologous region with *H. annuus* was the shortest, spanning 150,906 bp and accounting for 50.76%. Notably, reciprocal alignment analysis revealed variation in homologous coverage ratios among species. The coverage of *C. indicum* against *A. lactea* reached 78.53%, indicating a high degree of syntenic correspondence between the two species. Syntenic blocks were predominantly distributed as short, discrete fragments with both forward and reverse orientations, indicating extensive structural rearrangements and inversions in the mitochondrial genomes of these species.

### 3.7 Identification of mitochondrial plastid DNAs (MTPTs)

**Figure 9:**
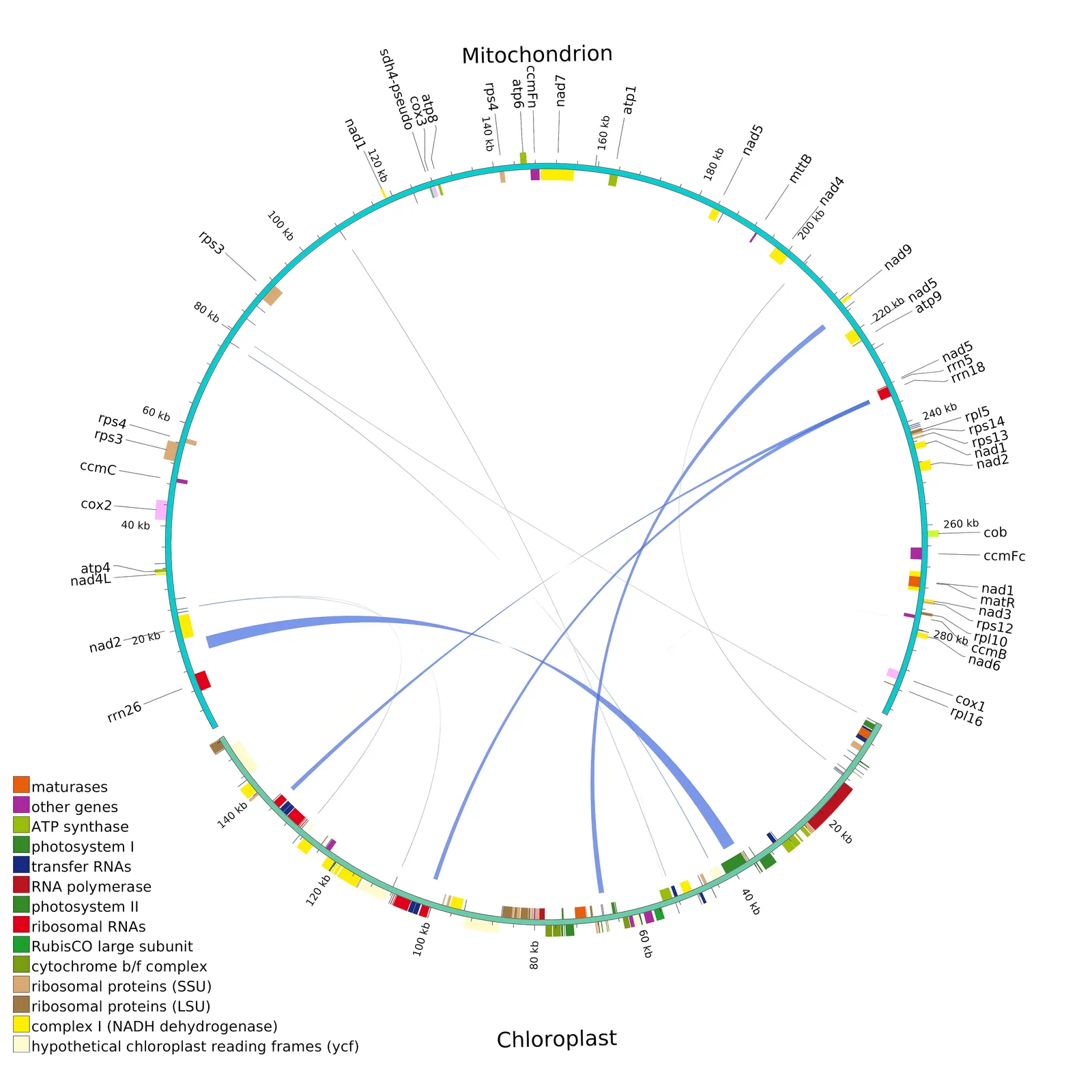
Homologous fragments between the chloroplast and mitochondrial genomes. Chloroplast: chloroplast genome; other tracks: mitochondrial genome. Same-color blocks: genes from the same complex. Outer/inner blocks: forward/reverse strand genes. Connecting lines: homologous sequences.

To reliably identify sequence similarities and eliminate random matches, the mitochondrial and chloroplast genomes of *A. lactea* were aligned using BLASTN with stringent E-value parameters. Consequently, 11 chloroplast-derived homologous fragments were identified within the mitochondrial genome, with sequence identities ranging from 73.09% to 100%. The total non-redundant length of these inserted fragments was 4,981 bp, accounting for 1.68% of the entire mitochondrial genome. The lengths of these fragments varied from 28 to 2,559 bp, with the number of mismatches ranging from 0 to 183. These transferred fragments are referred to as mitochondrial plastid DNAs (MTPTs) (**Supplementary Table 7**; **Figure 9**). The longest transferred fragment, 2,559 bp in length, originated from the chloroplast psaB and psaA genes and was integrated into the intergenic spacer region of the mitochondrial genome. Among all transferred fragments, five chloroplast tRNA genes, namely trnW-CCA, trnN-GTT, trnH-GTG, trnM-CAT, and trnD-GTC, that migrated to the mitochondrion maintained their structural and functional integrity. Conversely, the remaining sequences, which included fragments of protein-coding genes such as *atpA*, *atpB*, *ycf3*, and *ndhA*, as well as the ribosomal RNA gene *rrn18*, were primarily scattered across intergenic spacer regions and had completely lost their original coding functions.

### 3.8 Phylogenetic analysis

**Figure 10:**
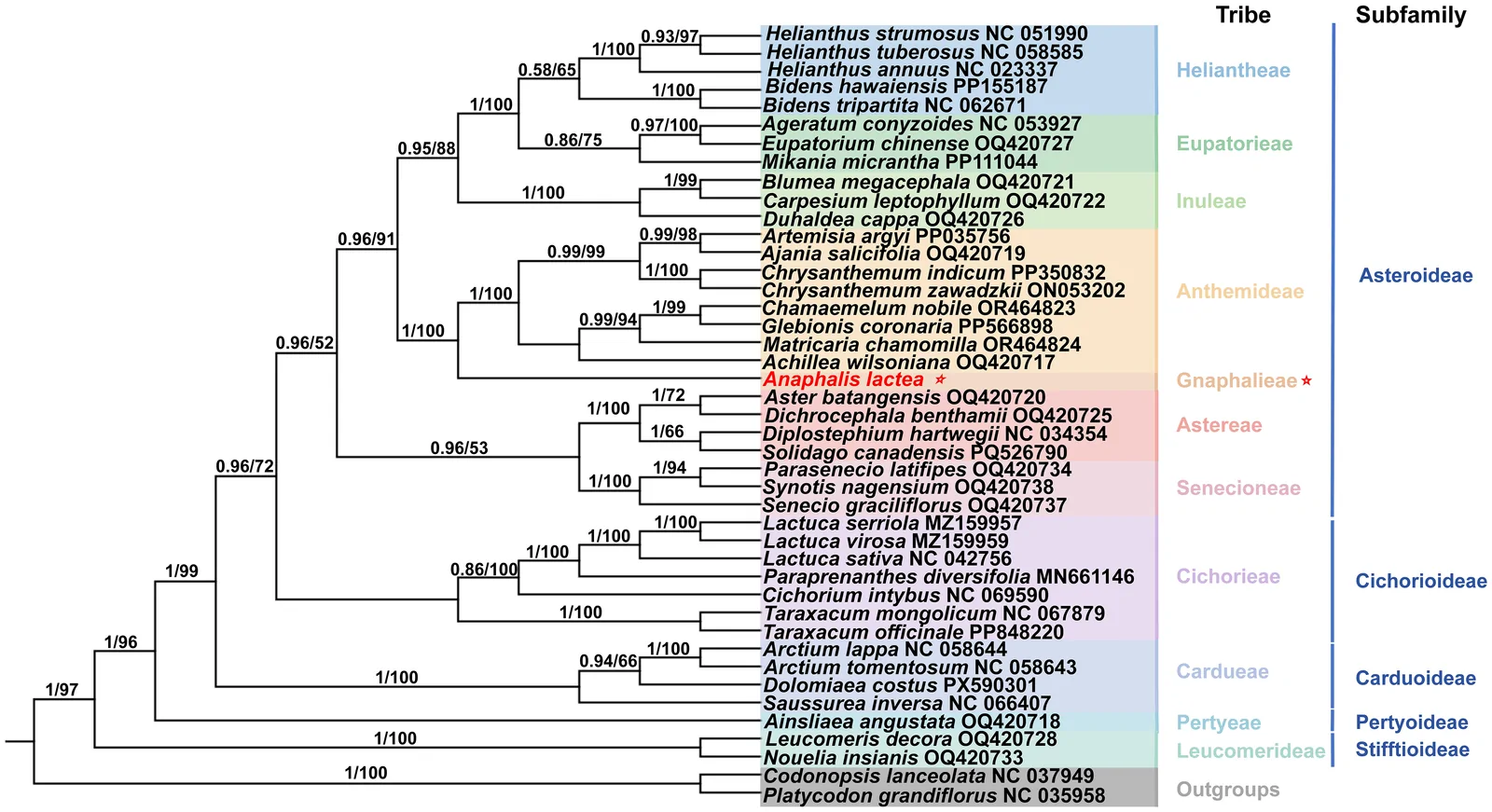
Phylogenetic tree of 41 representative Asteraceae species and two outgroup species based on 30 mitochondrial protein-coding genes. The tree was constructed using Bayesian inference (BI) and maximum likelihood (ML) methods. Bootstrap support values (BS) and posterior probabilities (PP) are shown at each node.

Bayesian inference (BI) and maximum likelihood (ML) phylogenetic trees were reconstructed based on 30 mitochondrial protein-coding genes (PCGs) from 41 representative Asteraceae species, with *Codonopsis lanceolata* and *Platycodon grandiflorus* designated as outgroups. The resulting topologies were highly congruent between the two methods, with posterior probabilities (PP) and bootstrap support (BS) values annotated at each node (**Figure 10**).

The phylogenetic tree grouped the selected Asteraceae species into five subfamilies, namely Asteroideae, Cichorioideae, Carduoideae, Pertyoideae, and Stifftioideae, each of which formed a strongly supported monophyletic clade. The overall topology was highly congruent with the currently accepted classification system of Asteraceae. The target species, *A. lactea*, resolved as a distinct clade representing Gnaphalieae and formed a sister-group relationship with Anthemideae (PP = 1.00, BS = 100). The topology further showed that this clade clustered with Astereae and Senecioneae, forming a strongly supported higher-level lineage. Additionally, Heliantheae, Eupatorieae, and Inuleae occupied relatively basal positions within Asteroideae.

### 3.9 Comparative analysis of mitochondrial protein-coding gene retention among representative Asteraceae species

**Figure 11:**
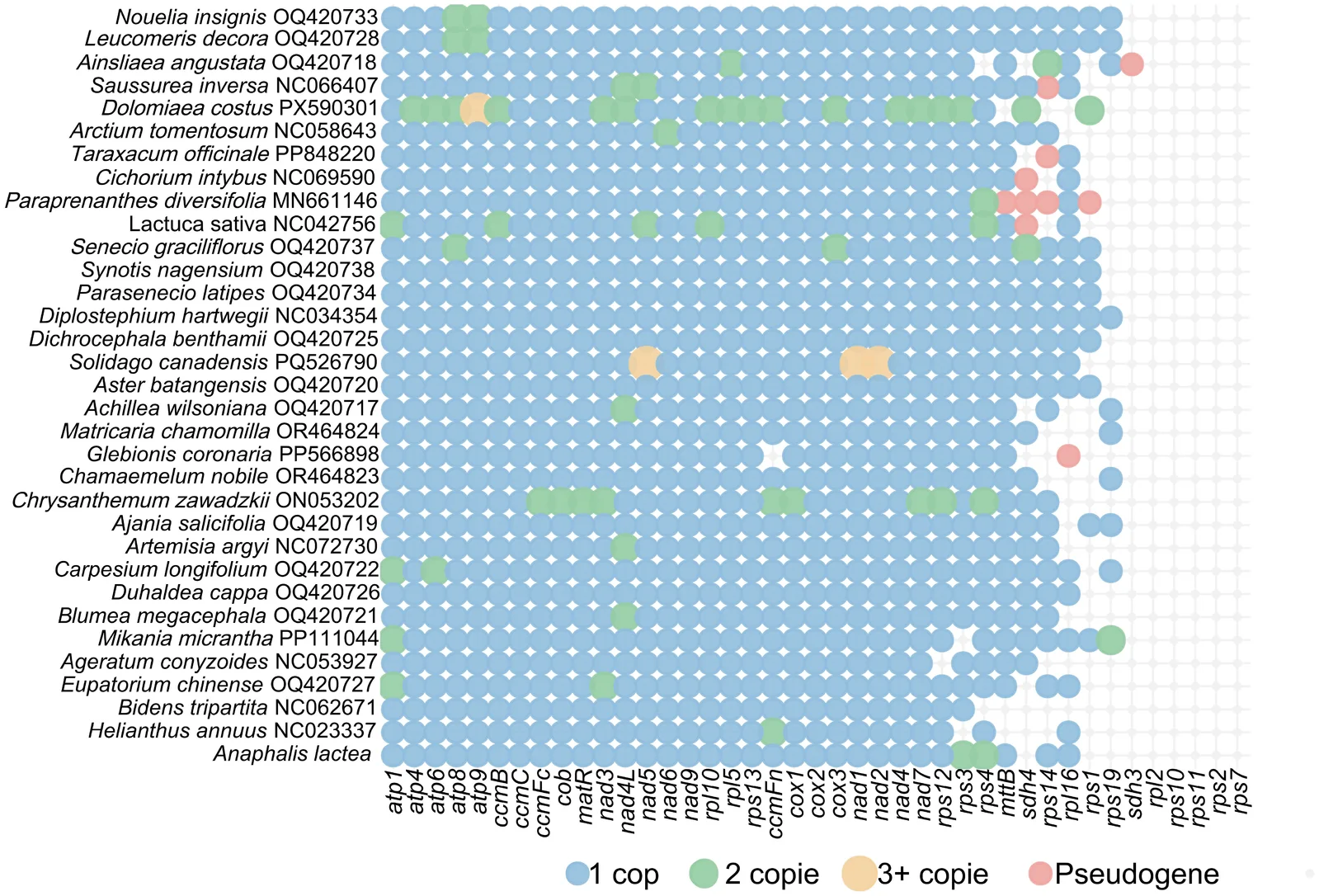
Comparison of mitochondrial core gene content among 33 Asteraceae species. A total of 41 core protein-coding genes are included. Different colors represent distinct gene statuses.

To investigate the retention patterns of 41 PCGs in Asteraceae, 33 representative species covering the major phylogenetic clades were selected (**Figure 11**). The core respiratory chain genes involved in oxidative phosphorylation, including the *atp*, *cox*, and *cob* gene families as well as most nad genes, are highly conserved among the studied Asteraceae species, exhibiting minimal copy number variation and predominantly existing as single-copy genes. In contrast, ribosomal protein genes (RPGs) and succinate dehydrogenase genes (sdh) exhibited substantial copy number variation and lineage-specific gene loss, indicating a highly dynamic evolutionary pattern. Notably, genes such as *rps1*, *rps2*, *rps7*, *rps10*, *rps11*, *rps14*, *rps19*, *rpl2*, *sdh3*, and *sdh4* frequently underwent loss or pseudogenization across multiple species; in particular, *sdh3*, *rpl2*, *rps10*, *rps11*, *rps2*, and *rps7* were absent in all examined taxa. Furthermore, copy number expansion was also observed in certain genes. For instance, duplicated copies of *atp1*, *atp6*, *atp8*, *nad4L*, *ccmB*, *ccmFn*, and *rps4* were detected in several species, whereas triplications were occasionally observed in genes such as *atp5*, *atp9*, *nad1*, and *nad2*.

## 4 Discussion

### 4.1 Structural dynamics and functional conservation of the mitochondrial genome

In this study, the complete mitochondrial genome of *A. lactea* was assembled into a single circular molecule, with a total length of 297,265 bp and a GC content of 44.71%. This genome size is intermediate among sequenced Asteraceae mitochondrial genomes, whereas the GC content remains relatively stable, suggesting that although plant mitochondrial genomes exhibit high structural plasticity, their base composition is under strong evolutionary constraint (Liu et al., 2023). The *A. lactea* mitochondrial genome encodes 32 functional genes involved in energy metabolism and protein synthesis, most of which are core respiratory chain genes. This indicates that the oxidative phosphorylation system remains the most functionally constrained component of plant mitochondrial genomes. Long-term exposure to high-altitude, hypoxic, and high-UV environments imposes continuous selection pressure on ATP synthesis efficiency. Consequently, the high conservation of core respiratory chain genes is crucial for maintaining stable mitochondrial energy metabolism and normal cellular physiological functions (Farhat et al., 2019; Yang et al., 2024; Zhang et al., 2023).

Meanwhile, several genes, including *rps1*, *rps2*, *rps11*, and *sdh3*, are missing from the assembly. Such gene loss is common in plant mitogenome evolution, as seen in *Mikania cordata* (Wang et al., 2024d) and *Populus kangdingensis* (Huang et al., 2025). This suggests that these taxa may have undergone convergent gene loss or transfer to the nuclear genome. The functional roles of these lost genes are typically replaced by nuclear counterparts to maintain normal protein expression (Adams et al., 2001). Furthermore, genes such as *nad1*, *nad2*, *nad5*, and *nad7* retain multiple introns. This is closely associated with complex RNA splicing and post-transcriptional regulation mechanisms in angiosperms (Brown et al., 2014). Additionally, genes like *rps3*, *rps4*, and *trnM-CAT* exhibit multiple copies. The multicopy status of tRNA genes likely helps maintain mitochondrial translation efficiency (Zhang et al., 2024), while the four copies of *trnM-CAT* may be linked to repeat-mediated genomic recombination events.

Although plant mitochondrial genomes have a strong capacity to integrate exogenous DNA (Richardson and Palmer, 2007), the proportion of mitochondrial plastid DNAs (MTPTs) in *A. lactea* is only 1.68%. This suggests that *A. lactea* may strictly limit the retention of exogenous DNA within coding regions to maintain the structural stability of core functional genes. This proportion is significantly lower than in most angiosperms, such as *Ipomoea batatas* (7.35%) (Yang et al., 2022), *Limonia acidissima* (15.75%) (Nam et al., 2024), *Paphiopedilum micranthum* (10.34%) (Yang et al., 2023) and contrasts sharply with the large-scale sequence transfer observed in *Hippophae tibetana* (17.97%), which shares a similar high-altitude habitat. This discrepancy indicates that even under comparable high-altitude selection pressures, mitochondrial genomes of different species may follow divergent evolutionary trajectories. In contrast, *H. tibetana* tends to promote genomic rearrangement through large-scale exogenous integration to adapt to its environment (Zeng et al., 2024), whereas *A. lactea* adopts a more conservative and stable strategy, ensuring stable operation of the oxidative phosphorylation system under extreme conditions by minimizing interference from exogenous sequences in coding regions.

Further analysis revealed that MTPT fragments primarily originated from chloroplast energy-conversion-related gene regions, involving functional modules such as photosystem I, the cytochrome b6f complex, ATP synthase, and the NDH complex, corresponding to the *psa*, *pet*, *atp*, and *ndh* gene families, respectively. This indicates a biased pattern in their origin. Consistent with general patterns, transferred tRNA genes remained largely intact to maintain mitochondrial translational function. Conversely, most PCGs retained only partial, truncated sequences and lost their original functions, and were predominantly located in intergenic spacers (IGSs). This phenomenon further confirms that mitochondria possess mechanisms for the “selective acceptance” and species-specific retention of foreign DNA fragments (Timmis et al., 2004; Smith and Keeling, 2015).

### 4.2 Repeat sequence-driven structural remodeling and dynamic genomic evolution

Repetitive sequences are key structural elements driving genome expansion and structural variation. They also mediate the regulation of gene expression (Mower et al., 2012). In this study, 271 repeat sequences were identified, with DSRs as the predominant type. This distribution pattern is similar to that observed in other Asteraceae species, indicating a degree of conservation in repeat sequence composition within this taxon. However, *A. lactea* contains five long repeat sequences exceeding 1 kb in length, the longest reaching 8,367 bp, demonstrating high internal structural plasticity. These long repeats likely trigger frequent sequence rearrangements and structural reconfiguration via non-allelic homologous recombination, thereby generating subgenomic circular molecules and structural heterogeneity. In addition, 183 medium-sized repeats (50-1,000 bp) were identified. Although such sequences are generally considered to have lower recombination activity and to undergo largely irreversible recombination events (Unseld et al., 1997), the recombination they mediate may still be influenced by environmental stimuli and developmental signals, thereby contributing to phenotypic plasticity (Mackenzie and Kundariya, 2020). Furthermore, SSRs are predominantly tetranucleotides with a distinct A/T bias. This bias is likely associated with mutational preferences during DNA replication and repair processes (Alan, 2013). Notably, 90.67% of SSR loci are located within genic regions. Additionally, TSRs are significantly enriched in the *nad2* gene region. Given that Complex I serves as the entry point of oxidative phosphorylation and a major site of electron leakage and reactive oxygen species (ROS) accumulation under hypoxic conditions (Galkin et al., 2009), repeat enrichment in the *nad2* region may not only reflect high local structural plasticity but also modulate DNA conformation, RNA processing, or gene expression, thereby enhancing mitochondrial responsiveness to environmental stress (Lupold et al., 1999; Sayyed et al., 2024).

Synteny analysis further revealed extensive inversion and rearrangement events between *A. lactea* and other Asteraceae species, with homologous blocks discretely distributed, indicating widespread structural remodeling of plant mitochondrial genomes throughout their evolutionary history. Notably, despite pervasive structural rearrangements across taxa, *A. lactea* and *C. indicum* exhibited the highest syntenic coverage, demonstrating that closely related species retain a high level of mitochondrial structural conservation. This result further supports the notion that repeat-mediated homologous recombination is a key driver of plant mitochondrial genome evolution, while highlighting that substantial structural conservation can be maintained among closely related lineages.

### 4.3 Potential regulatory role of RNA editing on mitochondrial functional stability

RNA editing is a key post-transcriptional modification in plant organellar gene expression, primarily involving cytidine-to-uridine (C-to-U) conversion (Ichinose and Sugita, 2016). Beyond transcript maturation, RNA editing serves as an important post-transcriptional regulatory mechanism in plant responses to abiotic stress. It modulates organellar protein function without altering genomic DNA sequences, thereby enhancing plant responsiveness to environmental fluctuations (Hu et al., 2024; Zu et al., 2023). In this study, all predicted RNA editing sites were non-synonymous, indicating that RNA editing regulates mitochondrial protein function primarily by altering amino acid sequences (Nishikura, 2010). These RNA editing sites preferentially occurred at the second codon position and promoted amino acid changes from hydrophilic to hydrophobic residues. This pattern is highly consistent with findings in *Phlomoides rotata* (Liu et al., 2025) and *Myricaria elegans* (Li et al., 2024), suggesting that this editing mode represents a conserved mitochondrial strategy for maintaining protein function in plants.

Notably, RNA editing sites were primarily enriched in respiratory chain genes, such as *nad*, *ccm*, *cox*, and *atp* families. These genes encode key components of the oxidative phosphorylation system, including Complex I, Complex IV, and ATP synthase, thereby directly affecting electron transport efficiency and ATP synthesis (Braun et al., 2014). Previous studies have shown that pentatricopeptide repeat (PPR) protein-mediated RNA editing of genes such as *nad2*, *nad*6, and *rps4* is essential for normal plant growth under low-temperature conditions (Zu et al., 2023), further highlighting RNA editing as a key regulatory mechanism for maintaining mitochondrial functional homeostasis. Additionally, this study identified *ccmFn* as the gene with the highest number of RNA editing sites within the *ccm* family. Given that previous research indicates its RNA editing levels can be modulated by environmental stress, it is likely that the cytochrome *c* maturation system also participates in plant stress responses (Ramadan et al., 2023).Moreover, the high frequency of leucine (Leu) and serine (Ser) substitutions may contribute to structural stability of respiratory chain proteins; specifically, the hydrophobic nature of Leu facilitates tighter embedding of key transmembrane proteins, such as Complex I, into the mitochondrial inner membrane lipid bilayer (Baumann and Zerbe, 2024).

Collectively, based on the strong enrichment of RNA editing sites in respiratory chain genes, their exclusively non-synonymous nature, and the resulting increase in hydrophobicity, we propose that *A. lactea* may employ RNA editing to enhance structural plasticity and functional stability of respiratory chain proteins while maintaining a highly conserved core mitochondrial genome, thereby sustaining oxidative phosphorylation under alpine conditions. This synergistic strategy, coupling genomic conservation with post-transcriptional regulation to enhance functional plasticity, may represent a key molecular mechanism underlying long-term adaptation to alpine habitats. It should be noted that the RNA editing sites identified in this study were based primarily on bioinformatic predictions, and experimental validation using Sanger sequencing and PCR is required.

### 4.4 Selective pressure shapes mitochondrial gene conservation and adaptive divergence

Codon usage bias is closely associated with gene expression efficiency, environmental adaptation, and species evolution (Sharp and Li, 1987). The overall codon usage bias in the mitochondrial genome of *A. lactea* was relatively weak. However, neutrality plot and ENC-plot analyses indicated that codon usage patterns were primarily shaped by natural selection. The observed ENC values of most genes were lower than the theoretical expected curve, suggesting that key functional genes are subjected to strong selective constraints during translation. Similar patterns have also been reported in *Angelica biserrata* (Wang et al., 2024b) and *Medicago* species (Shen et al., 2025). Natural selection-driven codon preference may reduce translation errors and improve translational efficiency, thereby facilitating the rapid and accurate expression of respiratory chain-related genes under extreme environmental stress. Consequently, this may contribute to the stability of mitochondrial energy metabolism in *A. lactea*.

Ka/Ks and Pi analyses are widely used to evaluate the evolutionary characteristics of mitochondrial genes (Yang and Bielawski, 2000). In this study, most protein-coding genes exhibited Ka/Ks ratios lower than 1, indicating that these genes were primarily subjected to purifying selection during evolution. Notably, core respiratory chain genes such as *cox1*, *cox3*, and *atp9* exhibited Ka/Ks values approaching zero, reflecting their high level of sequence conservation across species. This pattern is consistent with the strong conservation of respiratory chain genes reported in most plant mitochondrial genomes (Jin et al., 2021; Li et al., 2025). In the alpine subnival plant *Chorispora bungeana*, early cold stress maintains continuous mitochondrial electron transport chain activity, stabilizes mitochondrial membrane potential, and suppresses excessive ROS accumulation through rapid transitions in the ubiquinone redox state. This provides physiological support for the evolutionary constraints reflected by the high conservation of core respiratory chain genes observed in this study (Chang et al., 2006). In contrast, genes such as *nad3* and *atp8* exhibited relatively high evolutionary rates, suggesting their potential for functional divergence. Pi analysis showed that most gene sequences were relatively conserved. Conversely, genes such as *rps14*, *cox2*, and *ccmFc* exhibited relatively high nucleotide diversity, suggesting that these genes may possess greater evolutionary plasticity.

The *ccmFn* and *ccmFc* genes are involved in cytochrome *c* maturation and are closely associated with mitochondrial electron transport chain assembly and electron transfer efficiency. Therefore, site-specific selective pressure analyses were further performed on these two genes (Yong et al., 2024; Meyer et al., 2004). The results showed that *ccmFc* exhibited significant signals of positive selection in multiple model comparisons (*P* < 0.001). Three-dimensional structural modeling further identified the locations of the three positively selected sites, 285L, 303Y, and 378T, which were clustered within the β-sheet region of the protein core functional domain. This region forms an important structural component of the heme-binding pocket. Amino acid substitutions at these sites induced distinct localized conformational changes (Brausemann et al., 2021; Biancalana et al., 2015). Such structural remodeling of the *ccmFc* protein may optimize heme-binding affinity and enhance electron transfer efficiency (Mavridou et al., 2013). In the high-altitude-adapted *Peromyscus maniculatus*, enhanced COX activity under hypoxic conditions depends on intact heme assembly mediated by the ccm system. Moreover, positively selected sites are frequently enriched in proteins involved in respiratory chain supercomplex assembly (Mahalingam, 2017). This mechanism may represent an important adaptive strategy evolved by *A. lactea* to cope with alpine hypoxia and cold stress. Ultimately, this adaptation may help maintain mitochondrial respiratory chain energy homeostasis.

### 4.5 Phylogenetic incongruence and evolutionary complexity resolved by core PCGs

Phylogenetic analysis based on 30 mitochondrial protein-coding genes (PCGs) showed that *A. lactea* formed an independent, strongly supported clade among the selected representative species and that Gnaphalieae and Anthemideae form a sister group. Currently, phylogenetic studies of the tribe Gnaphalieae rely mainly on nuclear and chloroplast genomes, dividing it into two major lineages: the basal Relhaniinae, mainly endemic to Africa, and the core Gnaphaliinae, which contains more than 90% of species diversity (Xu et al., 2024; Lao et al., 2024; Hoson et al., 2022; Smissen et al., 2020).

The Gnaphalieae + Anthemideae topology obtained in this study differs from previous nuclear genomic studies, which support Gnaphalieae and Astereae as sister groups. This inconsistency is not isolated but reflects the cyto-nuclear discordance commonly observed in rapidly radiating lineages of the Asteraceae. A phylogenetic tree based on 74 chloroplast genes supported Gnaphalieae + (Anthemideae + Astereae), whereas a nuclear phylogenomic analysis based on nearly one thousand gene loci supported Gnaphalieae + Astereae, indicating a clear topological conflict (Watson et al., 2020). In addition, comparative studies based on complete chloroplast genomes generally support Astereae and Anthemideae as sister groups, with Gnaphalieae positioned outside them, further indicating that different genomic compartments may retain distinct phylogenetic signals (Fu et al., 2016; Panero and Crozier, 2016; Abdullah et al., 2021).

This topological conflict may not be solely explained by differences among genomic systems. Watson et al. further inferred that ancient reticulate evolutionary events may have occurred between Anthemideae and Gnaphalieae, and that reticulate signals are also present within Astereae, Anthemideae, and Senecioneae (Watson et al., 2020). This indicates that the evolutionary history of rapidly radiating Asteraceae lineages is not a simple bifurcating process but is shaped by processes such as ancient hybridization, introgression, and incomplete lineage sorting (ILS), resulting in different genomes recovering conflicting topologies. The Gnaphalieae + Anthemideae relationship recovered in this study may represent a subset of the phylogenetic signal underlying the complex evolutionary history of this group. Moreover, plant mitochondrial genomes are characterized by homologous recombination, structural rearrangements, and potential horizontal gene transfer (HGT), which may further increase phylogenetic signal complexity (Ye et al., 2024; Stiller, 2011). Future studies should integrate nuclear, chloroplast, and mitochondrial genomic data and apply approaches such as coalescent models, hybridization detection, and phylogenetic network analyses to further resolve the evolutionary history of the tribe Gnaphalieae and rapidly radiating Asteraceae lineages.

### 4.6 Potential links between mitochondrial genome evolution and adaptation to the extreme alpine environment

Previous studies have demonstrated that *A. lactea* adapts to the complex environmental stresses of the Qinghai-Xizang Plateau, including severe cold, hypoxia, and intense radiation, through diverse morphological and physiological mechanisms. These include adjusting leaf cuticular wax composition, modifying leaf functional traits, and coordinating resource allocation between vegetative and reproductive growth (Yao et al., 2022; Feng et al., 2025). The results of this study further indicate that the mitochondrial genome of *A. lactea* has not undergone extensive adaptive sequence divergence. Instead, despite overall high conservation, it responds to long-term high-altitude selection pressure through post-transcriptional regulation and localized gene-level adaptive evolution. First, core genes of the oxidative phosphorylation system, such as cox, nad, and atp, are under strong purifying selection, indicating that the maintenance of core respiratory chain function remains a key feature of mitochondrial genome evolution. Second, RNA editing sites are mainly enriched in respiratory chain genes such as *nad*, *ccm*, and *cox*, and are exclusively non-synonymous. Approximately half of these edits result in amino acid substitutions from hydrophilic to hydrophobic residues, suggesting that, under relatively conserved DNA sequences, post-transcriptional modifications may optimize the structure and function of respiratory chain complexes by altering protein physicochemical properties. In addition, a significant positive selection signal was detected only in *ccmFc*, with selected sites mainly distributed in key functional regions of the protein, suggesting that the cytochrome c maturation system may represent a relatively more evolutionarily plastic module within mitochondrial energy metabolism and may contribute to adaptation to long-term high-altitude stresses such as severe cold and hypoxia.

Overall, the evolutionary pattern of the *A. lactea* mitochondrial genome does not rely on extensive changes in protein-coding sequences but instead achieves long-term adaptation to complex high-altitude environments through RNA editing-mediated functional plasticity and localized adaptive evolution of a few key genes, while maintaining high conservation of the core energy metabolism system. This evolutionary strategy, balancing functional conservation and molecular plasticity, may provide a basis for maintaining mitochondrial functional homeostasis under conditions of severe cold, strong radiation, and large diurnal temperature variation. However, these conclusions are primarily based on comparative genomics and bioinformatic analyses. Future studies should integrate transcriptomic, proteomic, and functional approaches to further validate the underlying molecular mechanisms.

## 5 Conclusion

In this study, we assembled and comprehensively characterized the complete mitochondrial genome of *A. lactea*, systematically elucidating its genomic structure, repeat sequences, RNA editing, codon usage bias, selective pressures, and phylogenetic attributes. Our results demonstrate that this mitogenome exhibits an evolutionary pattern characterized by the coexistence of structural dynamism and functional conservation. While repeat sequences and RNA editing jointly enhance genomic plasticity, the high conservation of core respiratory chain genes, coupled with localized adaptive divergence in a few energy metabolism-related genes, likely maintains mitochondrial functional homeostasis under high-altitude hypoxic conditions. These findings deepen our understanding of mitogenome evolution and environmental adaptation in alpine plants, while providing valuable genomic resources and comparative benchmarks for future research on Asteraceae phylogenetics and organellar genome evolution.

## Data Availability

The datasets presented in this study can be found in online repositories. The names of the repository/repositories and accession number(s) can be found below: https://ngdc.cncb.ac.cn/gsa, CRA042929.
