# Supplementary material for: Mitochondrial genome of Anaphalis lactea from the Qinghai-Xizang Plateau: structural and evolutionary insights into alpine adaptation
Source: Front Plant Sci. 2026 Aug 6;17:1885043. doi: 10.3389/fpls.2026.1885043 (PMC13490082; doi:10.3389/fpls.2026.1885043)
Supplement: Supplementary file 1 [file Table1.docx]

Supplementary Material

Table S1. Functional classification and gene composition of protein-coding genes (PCGs) in the *Anaphalis lactea* mitogenome.

| **Functional category** | **Gene** | **Count** |
| --- | --- | --- |
| ATP synthase | atp1, atp4, atp6, atp8, atp9 | 5 |
| Cytochrome c biogenesis | ccmB, ccmC, ccmFc, ccmFn | 4 |
| Cytochrome c oxidase | cox1, cox2, cox3 | 3 |
| Cytochrome b | cob | 1 |
| NADH dehydrogenase | nad1, nad2, nad3, nad4, nad4L, nad5, nad6, nad7, nad9 | 9 |
| Ribosomal protein | rps3, rps4, rps12, rps13, rps14, rpl5, rpl10, rpl16 | 8 |
| Maturase-related | matR | 1 |
| Transport-related protein | mttB | 1 |
| **Total** |  | **34** |

Table S2. Dispersed repeats identified in the *Anaphalis lactea* mitogenome.

| **NO.** | **Size** | **Repeat sequence** | **Percent Matches** |
| --- | --- | --- | --- |
| 1 | 21 | CATAAAGAGGGCCTTAGAATT | 91 |
| 2 | 18 | GACTATGAAACAGATCGC | 81 |
| 3 | 71 | AAAGTTCTATCTTATAGAACTGCATAATGATACTCTCACTTTTCCCTTCGAAGAGCTGATCTTGAATTGAA | 88 |
| 4 | 71 | TGATACTTTCACTTTTCCCTTCGAAGAGCTGATCTTGAATTGAAAAAGTTCTATCTTATAGAACTGCATTT | 93 |
| 5 | 33 | TAGGGGGAGTATAAGTTAAAGGCGGGATTGTCT | 94 |
| 6 | 24 | AGGAAGTCGATTCTGTCTCACAGT | 95 |
| 7 | 20 | TCTAATCCTAGACTAAAGAA | 100 |
| 8 | 71 | AAAGTATCAAAATGCAGTTCTATAAGATAGAACTTTTTCAATGAAAGATCAACTCATCGAAGGGAAAAGTG | 93 |
| 9 | 71 | AAAGTATCAAAATGCAGCTATATAAGATAGAACTTTTTCAATGAAAGATCAACTCATCGAAGGGAAAAGTG | 88 |
| 10 | 15 | CCAAGTCAAGTTGAT | 100 |
| 11 | 15 | AAGAGCTACAGAAGG | 76 |
| 12 | 39 | AATATCATGATCGGGTCGACCAGGCCAGATCATGAGTGA | 95 |
| 13 | 16 | GTCAGTCCCTAACTAT | 100 |
| 14 | 71 | AAAGTTCTATCTTATAGAACTGCATAATGATACTCTCACTTTTCCCTTCGAAGAGCTGATCTTGAATTGAA | 88 |
| 15 | 71 | TGATACTTTCACTTTTCCCTTCGAAGAGCTGATCTTGAATTGAAAAAGTTCTATCTTATAGAACTGCATTT | 93 |
| 16 | 20 | CTTAACTGTTTTTCCCCTTAC | 91 |
| 17 | 15 | TCTTCTTAGGTGCTT | 72 |
| 18 | 12 | TCTTAGGTTTCT | 80 |
| 19 | 29 | TCTATTCTAAGCTAGGCAGTGTTTCTTCT | 100 |
| 20 | 71 | AAAGTATCAAAATGCAGTTCTATAAGATAGAACTTTTTCAATGAAAGATCAACTCATCGAAGGGAAAAGTG | 93 |
| 21 | 71 | AAAGTATCAAAATGCAGCTATATAAGATAGAACTTTTTCAATGAAAGATCAACTCATCGAAGGGAAAAGTG | 88 |
| 22 | 71 | AAAGTTCTATCTTATAGAACTGCATAATGATACTCTCACTTTTCCCTTCGAAGAGCTGATCTTGAATTGAA | 88 |
| 23 | 71 | TGATACTTTCACTTTTCCCTTCGAAGAGCTGATCTTGAATTGAAAAAGTTCTATCTTATAGAACTGCATTT | 93 |
| 24 | 29 | AGAAGAAACACTGCCTAGCTTAGAATAGA | 100 |
| 25 | 21 | TGGAGGAGAGCGACAGAGCTC | 82 |
| 26 | 17 | TATTAATAAGATAGGTT | 100 |
| 27 | 16 | CGCAGAAAGAGCATCC | 88 |
| 28 | 32 | TCTCACTCGGGAGAAGTAGTCCCCGGAAAGT | 90 |
| 29 | 18 | TAACTGCATACAAACAAA | 94 |
| 30 | 12 | AGAAACCTAAGA | 80 |

Table S3. Summary of amino acid property changes induced by RNA editing in the *Anaphalis lactea* mitogenome.

| **Type** | **RNA-editing** | **Number** | **Percentage** |
| --- | --- | --- | --- |
| hydrophilic-hydrophilic | CAC (H) => TAC (Y) | 7 |  |
|  | CAT (H) => TAT (Y) | 17 |  |
|  | CGC (R) => TGC (C) | 9 |  |
|  | CGT (R) => TGT (C) | 25 |  |
|  | total | 58 | 10.30% |
| hydrophilic-hydrophobic | ACA (T) => ATA (I) | 8 |  |
|  | ACC (T) => ATC (I) | 5 |  |
|  | ACG (T) => ATG (M) | 7 |  |
|  | ACT (T) => ATT (I) | 6 |  |
|  | CGG (R) => TGG (W) | 31 |  |
|  | TCA (S) => TTA (L) | 75 |  |
|  | TCC (S) => TTC (F) | 29 |  |
|  | TCG (S) => TTG (L) | 45 |  |
|  | TCT (S) => TTT (F) | 60 |  |
|  | total | 266 | 47.25% |
| hydrophilic-stop | CGA (R) => TGA (X) | 2 |  |
|  | total | 2 | 0.36% |
| hydrophobic-hydrophilic | CCA (P) => TCA (S) | 9 |  |
|  | CCC (P) => TCC (S) | 10 |  |
|  | CCG (P) => TCG (S) | 4 |  |
|  | CCT (P) => TCT (S) | 20 |  |
|  | total | 43 | 7.64% |
| hydrophobic-hydrophobic | CCA (P) => CTA (L) | 48 |  |
|  | CCC (P) => CTC (L) | 10 |  |
|  | CCC (P) => TTC (F) | 3 |  |
|  | CCG (P) => CTG (L) | 36 |  |
|  | CCT (P) => CTT (L) | 24 |  |
|  | CCT (P) => TTT (F) | 6 |  |
|  | CTC (L) => TTC (F) | 13 |  |
|  | CTT (L) => TTT (F) | 33 |  |
|  | GCA (A) => GTA (V) | 6 |  |
|  | GCC (A) => GTC (V) | 4 |  |
|  | GCG (A) => GTG (V) | 7 |  |
|  | GCT (A) => GTT (V) | 4 |  |
|  | total | 194 | 34.46% |
|  | All | 563 | 100% |

Table S4. Codon usage and relative synonymous codon usage (RSCU) of mitochondrial protein-coding genes in *Anaphalis lactea*.

| **AminoAcid** | **Symbol** | **Codon** | **No.** | **RSCU** |
| --- | --- | --- | --- | --- |
| * | Ter | UAA | 16 | 1.5 |
| * | Ter | UAG | 6 | 0.5625 |
| * | Ter | UGA | 10 | 0.9375 |
| A | Ala | GCA | 148 | 0.9533 |
| A | Ala | GCC | 146 | 0.9404 |
| A | Ala | GCG | 75 | 0.4831 |
| A | Ala | GCU | 252 | 1.6232 |
| C | Cys | UGC | 54 | 0.7883 |
| C | Cys | UGU | 83 | 1.2117 |
| D | Asp | GAC | 113 | 0.6975 |
| D | Asp | GAU | 211 | 1.3025 |
| E | Glu | GAA | 285 | 1.3971 |
| E | Glu | GAG | 123 | 0.6029 |
| F | Phe | UUC | 277 | 0.8616 |
| F | Phe | UUU | 366 | 1.1384 |
| G | Gly | GGA | 237 | 1.4024 |
| G | Gly | GGC | 87 | 0.5148 |
| G | Gly | GGG | 134 | 0.7929 |
| G | Gly | GGU | 218 | 1.2899 |
| H | His | CAC | 53 | 0.453 |
| H | His | CAU | 181 | 1.547 |
| I | Ile | AUA | 217 | 0.8623 |
| I | Ile | AUC | 206 | 0.8185 |
| I | Ile | AUU | 332 | 1.3192 |
| K | Lys | AAA | 238 | 1.199 |
| K | Lys | AAG | 159 | 0.801 |
| L | Leu | CUA | 166 | 0.9522 |
| L | Leu | CUC | 108 | 0.6195 |
| L | Leu | CUG | 88 | 0.5048 |
| L | Leu | CUU | 226 | 1.2964 |
| L | Leu | UUA | 258 | 1.4799 |
| L | Leu | UUG | 200 | 1.1472 |
| M | Met | AUG | 266 | 1 |
| N | Asn | AAC | 104 | 0.671 |
| N | Asn | AAU | 206 | 1.329 |
| P | Pro | CCA | 157 | 1.1523 |
| P | Pro | CCC | 97 | 0.7119 |
| P | Pro | CCG | 92 | 0.6752 |
| P | Pro | CCU | 199 | 1.4606 |
| Q | Gln | CAA | 210 | 1.5 |
| Q | Gln | CAG | 70 | 0.5 |
| R | Arg | AGA | 138 | 1.3869 |
| R | Arg | AGG | 76 | 0.7638 |
| R | Arg | CGA | 133 | 1.3367 |
| R | Arg | CGC | 58 | 0.5829 |
| R | Arg | CGG | 73 | 0.7337 |
| R | Arg | CGU | 119 | 1.196 |
| S | Ser | AGC | 97 | 0.6319 |
| S | Ser | AGU | 170 | 1.1075 |
| S | Ser | UCA | 177 | 1.1531 |
| S | Ser | UCC | 139 | 0.9055 |
| S | Ser | UCG | 121 | 0.7883 |
| S | Ser | UCU | 217 | 1.4137 |
| T | Thr | ACA | 130 | 1.0236 |
| T | Thr | ACC | 133 | 1.0472 |
| T | Thr | ACG | 60 | 0.4724 |
| T | Thr | ACU | 185 | 1.4567 |
| V | Val | GUA | 176 | 1.1636 |
| V | Val | GUC | 116 | 0.7669 |
| V | Val | GUG | 137 | 0.9058 |
| V | Val | GUU | 176 | 1.1636 |
| W | Trp | UGG | 146 | 1 |
| Y | Tyr | UAC | 67 | 0.4512 |
| Y | Tyr | UAU | 230 | 1.5488 |

Table S5. Codon usage and nucleotide composition of protein-coding genes in the *Anaphalis* *lactea* mitogenome.

| **Gene** | **GC1** | **GC2** | **GC3** | **GC12** | **ENC** | **GCall** | **GC3s** | **codon.no.** |
| --- | --- | --- | --- | --- | --- | --- | --- | --- |
| nad2 | 41.51 | 43.97 | 34.15 | 42.74 | 52.56 | 39.88 | 14.55% | 506 |
| nad4L | 39.6 | 40.59 | 30.69 | 40.1 | 45.33 | 36.96 | 13.59% | 296 |
| atp4 | 47.4 | 42.19 | 40.62 | 44.79 | 61 | 43.40 | 20.24% | 74 |
| cox2 | 53.08 | 38.08 | 28.85 | 45.58 | 48.95 | 40.00 | 15.12% | 235 |
| ccmC | 45.82 | 47.01 | 35.86 | 46.41 | 50.69 | 42.90 | 33.05% | 616 |
| rps3 | 43.07 | 40.4 | 44.24 | 41.74 | 57.92 | 42.57 | 13.89% | 257 |
| rps4 | 38.31 | 42.37 | 33.56 | 40.34 | 48.86 | 38.08 | 11.39% | 384 |
| nad1 | 53.37 | 41.72 | 29.75 | 47.55 | 50.54 | 41.61 | 17.93% | 150 |
| cox3 | 53.38 | 43.98 | 36.09 | 48.68 | 56.8 | 44.48 | 17.74% | 494 |
| atp8 | 35.62 | 40.62 | 48.12 | 38.12 | 48.9 | 41.45 | 12.66% | 494 |
| atp6 | 40.52 | 33.77 | 31.17 | 37.14 | 52.97 | 35.15 | 10.74% | 527 |
| ccmFn | 51.14 | 47.64 | 42.03 | 49.39 | 57.7 | 46.94 | 33.82% | 264 |
| nad7 | 56.2 | 45.57 | 31.65 | 50.89 | 50.2 | 44.47 | 25.13% | 707 |
| atp1 | 56.95 | 42.07 | 34.05 | 49.51 | 53.22 | 44.36 | 25.20% | 212 |
| nad5 | 43.37 | 44.41 | 35.32 | 43.89 | 52.11 | 41.03 | 14.31% | 676 |
| mttB | 45.97 | 42.74 | 45.97 | 44.35 | 54.03 | 44.89 | 26.49% | 150 |
| nad4 | 44.56 | 38.1 | 35.89 | 41.33 | 50.58 | 39.52 | 15.15% | 494 |
| nad9 | 51.31 | 41.36 | 34.55 | 46.34 | 58.11 | 42.41 | 21.88% | 191 |
| atp9 | 52 | 53.33 | 34.67 | 52.67 | 43.37 | 46.67 | 30.67% | 74 |
| rpl5 | 48.94 | 36.7 | 40.43 | 42.82 | 57.35 | 42.02 | 11.23% | 186 |
| rps14 | 46.3 | 40.74 | 50 | 43.52 | 61 | 45.68 | 25.74% | 100 |
| rps13 | 49.57 | 41.03 | 23.08 | 45.3 | 36.61 | 37.89 | 21.01% | 118 |
| cob | 51.53 | 41.07 | 32.91 | 46.3 | 53.1 | 41.84 | 17.66% | 384 |
| ccmFc | 49.66 | 43.74 | 42.37 | 46.7 | 59.04 | 45.26 | 37.73% | 439 |
| matR | 52.9 | 45.27 | 57.77 | 49.09 | 58.05 | 51.98 | 35.31% | 707 |
| nad3 | 43.7 | 44.54 | 35.29 | 44.12 | 49.99 | 41.18 | 13.56% | 117 |
| rps12 | 53.97 | 49.21 | 28.57 | 51.59 | 47.53 | 43.92 | 26.40% | 124 |
| rpl10 | 47.85 | 42.94 | 36.81 | 45.4 | 60.06 | 42.53 | 21.57% | 152 |
| ccmB | 45.89 | 46.38 | 32.85 | 46.14 | 52.21 | 41.71 | 35.05% | 213 |
| nad6 | 43.69 | 34.81 | 38.91 | 39.25 | 54.99 | 39.14 | 10.33% | 212 |
| cox1 | 48.11 | 46.21 | 36.55 | 47.16 | 54.05 | 43.62 | 21.02% | 527 |
| rpl16 | 48.15 | 55.56 | 35.19 | 51.85 | 59.23 | 46.30 | 28.30% | 52 |

Table S6. Selection pressure analysis of the mitochondrial ccmFc gene in *Anaphalis lactea* using site models and branch-site models in PAML CodeML.

| **Model** | **Parameters** | **lnL** | **parameter estimates** | **Model comparison** | **LRT P-value** | **BEB** |
| --- | --- | --- | --- | --- | --- | --- |
| Points Model |  |  |  |  |  |  |
| M0 | 13 | -2423.878809 | ω= 0.88261 |  |  |  |
| M3 | 17 | -2423.244736 | p0=0.56868,ω0=0.00000 | M3&M0 | 0.866756384 | -- |
|  |  |  | p1=0.16591,ω1=0.00000 |  |  |  |
|  |  |  | p2=0.26541,ω2=3.39682 |  |  |  |
| M1a | 14 | -2423.741915 | p0=0.21624,ω0=0.00000 |  |  |  |
|  |  |  | p1=0.78376,ω1=1.00000 |  |  |  |
| M2a | 16 | -2423.244736 | p0=0.73458,ω0=0.00000 | M2a&M1a | 0.608244098 | -- |
|  |  |  | p1=0.00000,ω1=1.00000 |  |  |  |
|  |  |  | p2=0.26541,ω2=3.39683 |  |  |  |
| M7 | 14 | -2423.808299 | p=0.13084,q=0.03272 |  |  |  |
| M8 | 16 | -2423.244736 | p0=0.73458,p=0.01149,q=2.16337 |  |  |  |
|  |  |  | p1=0.26542,ω=3.39698 |  |  |  |
| M8a | 15 | -2423.741915 | p0=0.21618,p=0.00549,q=2.01322 | M8&M7 | 0.569177467 | -- |
|  |  |  | p1=0.78382,ω=1.00000 | M8&M8a | 0.318679569 | -- |
| Branch-site model |  |  |  |  |  |  |
| Model A null | 15 | -1746.985484 | Class0:f=0.00000,ω0=0.00000,ω1=0.00000 |  |  |  |
|  |  |  | Class1:f=0.00000,ω0=1.00000,ω1=1.00000 |  |  |  |
|  |  |  | Class2a:f=0.35593,ω0=0.00000,ω1=1.00000 |  |  |  |
|  |  |  | Class2b:f=0.64407,ω0=1.00000,ω1=1.00000 |  |  |  |
| Model A | 16 | -1746.985484 | Class0:f=0.00000,ω0=0.00000,ω1=0.00000 |  |  |  |
|  |  |  | Class1:f=0.00000,ω0=1.00000,ω1=1.00000 |  |  |  |
|  |  |  | Class2a:f=0.35445,ω0=0.00000,ω1=1.76204 |  |  |  |
|  |  |  | Class2b:f=0.64555,ω0=1.00000,ω1=1.76204 | Model A&Model A null | 0.584042954 |  |

Note: '--' indicates that the LRT (Likelihood Ratio Test) is not significant. The null hypothesis model is accepted, and no selection signal exists.

Table S7. Homologous fragments transferred from chloroplast to mitochondrial genome (MTPTs) in *Anaphalis lactea*.

| **Length (bp)** | **Identity (%)** | **Mismatches** | **Bitscore** | **Gene**  **(chloroplast)** | **Gene**  **(mitochondrion)** |
| --- | --- | --- | --- | --- | --- |
| 2,559 | 98.20 | 33 | 4,458 | psaB (partial),psaA (partial) | — |
| 1,121 | 78.23 | 158 | 640 | petL, petG, trnW-CCA, trnP-UGG | trnW-CCA |
| 892 | 73.09 | 183 | 265 | rrn16S (partial) | rrn18 (partial) |
| 892 | 73.09 | 183 | 265 | rrn16S (partial) | rrn18 (partial) |
| 143 | 86.01 | 9 | 143 | ycf3 (partial) | — |
| 84 | 96.43 | 2 | 137 | trnN-GUU | trnN-GTT |
| 84 | 96.43 | 2 | 137 | trnN-GUU | trnN-GTT |
| 80 | 97.50 | 2 | 137 | trnH-GUG | trnH-GTG |
| 79 | 96.20 | 3 | 130 | trnM-CAU | trnM-CAT |
| 88 | 92.05 | 7 | 124 | trnD-GUC | trnD-GTC |
| 28 | 100 | 0 | 52.8 | atpB (partial) | — |
